# Ιdentification of SQ109 analogs with enhanced antimicrobial activity against methicillin-resistant *Staphylococcus aureus*

**DOI:** 10.1128/aac.01545-25

**Published:** 2026-02-18

**Authors:** Charilaos Dellis, George Laros, Kyriakos Georgiou, Liyang Zhang, Lewis Oscar Felix, Nikolas Naziris, Narchonai Ganesan, Jianhua Gu, Marianna Stampolaki, Costas Demetzos, Ioannis P. Papanastasiou, Biswajit Mishra, Antonios Kolocouris, Eleftherios Mylonakis

**Affiliations:** 1 Department of Medicine, Houston Methodist Hospital 828032, Houston, Texas, USA; 2 Laboratory of Medicinal Chemistry, Section of Pharmaceutical Chemistry, Department of Pharmacy, National and Kapodistrian University of Athens 69232, Athens, Greece; 3 Section of Pharmaceutical Technology, Department of Pharmacy, School of Health Sciences, National and Kapodistrian University of Athens 69232, Athens, Greece; 4 Electron Microscopy Core, Houston Methodist Academic Institute 167626 , Houston, Texas, USA; The Peter Doherty Institute for Infection and Immunity, Melbourne, Victoria, Australia

**Keywords:** SQ109, gram-positive, anti-staphylococcal, MRSA, persisters, *S. aureus*

## Abstract

The rise of antimicrobial resistance necessitates the development of novel or repurposed molecules with potent antibacterial properties. In this study, we evaluated the lipid-based antimicrobial candidate, SQ109, and 14 of its analogs for their efficacy against methicillin-resistant *Staphylococcus aureus* (MRSA). Analogs AK126 and AK127, featuring a bulky benzyl- or phenyl-substituent at the adamantyl C-2 position, respectively, exhibited the most potent antimicrobial activity with no detectable resistance development. To elucidate their mechanisms of action, we combined molecular dynamics simulations, fluorescence-based assays, and scanning electron microscopy. Our results showed that SQ109, AK126, and AK127 target the *S. aureus* membrane by disrupting the proton motive force and inducing membrane damage in a dose-dependent manner. Additionally, AK126 and AK127 showed activity against *S. aureus* persister cells and synergized with gentamicin to facilitate its uptake. Lastly, both analogs exhibited higher selectivity for negatively charged membranes over the largely zwitterionic epithelial membrane. While further optimization is needed, these findings highlight two new scaffolds as a basis for the development of new agents capable of combating difficult-to-treat MRSA infections.

## INTRODUCTION

Antimicrobial resistance (AMR) is an escalating global health crisis, posing a major socioeconomic burden (1, 2). As resistance to existing antibiotics continues to rise, it is projected that multidrug-resistant (MDR) pathogens will reach over 8 million deaths annually by 2050 (3). Among the MDR pathogens, methicillin-resistant *Staphylococcus aureus* (MRSA) remains a major global threat, responsible for severe and often life-threatening infections (2). Treating MRSA is particularly challenging because it is resistant to β-lactam antibiotics, the traditional first-line therapies for these infections (4). This resistance, combined with its activity to form biofilms and persist in various clinical environments, complicates treatment and contributes to high morbidity and mortality rates (5). While antibiotics such as the glycopeptide vancomycin (a cell wall-targeting antibiotic) and the lipopeptide daptomycin are available treatment options, the increasing prevalence of resistance and subsequent treatment failures has led to a dramatic rise in MRSA-attributable deaths, nearly doubling from approximately 57,000 in 1990 to almost 130,000 in 2021, underscoring the urgent need for novel antimicrobial agents (3, 6).

The bacterial envelope, which comprises the membrane and cell wall, is an attractive target for the treatment of MDR pathogens (7). Membrane-active agents offer several advantages as potential antibiotics, such as rapid killing, a low likelihood of resistance development, and the ability to effectively target and eradicate bacterial persister cells (8, 9). Moreover, the membrane composition of gram-positive bacteria differs from that of mammalian cells, consisting mostly of anionic phospholipids such as phosphatidylglycerol (PG) and cardiolipin (CL) (10, 11). Although naturally occurring, cationic antimicrobial peptides exploit these differences to selectively target bacteria (12, 13), many membrane-active agents still exhibit limited selectivity, leading to high toxicity toward mammalian cells (14).

The lipid-based *N*-geranyl-*Ν′*-(2-adamantyl)ethane-1,2-diamine (SQ109) is a promising antibacterial candidate that has made it to Phase II clinical trials for the treatment of patients with tuberculosis (15). SQ109 inhibits the growth of *Mycobacterium tuberculosis* by targeting the mycobacterial membrane protein large 3 (Mmpl3), a trehalose monomycolate transporter involved in cell wall biosynthesis (16). In addition, it has also demonstrated uncoupler activity by disrupting the proton motive force (PMF) (17–20). The PMF is a critical component for membrane integrity and ATP synthesis and consists of the electric potential (Δψ) and the transmembrane proton gradient (ΔpH) (21). Thus, SQ109 has also demonstrated strong antimicrobial activity against other bacterial and fungal species, as well as protozoa that lack the Mmpl3, possibly by disrupting the PMF (19, 22–25).

Previously, members of our group synthesized a set of SQ109 analogs with enhanced inhibitory activity against *M. tuberculosis* as well as *Bacillus subtilis* and against malaria parasites (26). As a continuation of our efforts to explore the activity of SQ109 and analogs against other pathogens, the present study aimed to evaluate the activity of SQ109 and its analogs bearing alkyl or aryl adducts at the adamantyl C-2 carbon against MRSA. In addition, we investigated the mechanism of action of these compounds, examined their antibiofilm potential, and tested their activity to kill persister cells. Finally, we evaluated their cytotoxic activity and the *in vivo* efficacy of the most potent analogs in a *Caenorhabditis elegans* infection model.

## MATERIALS AND METHODS

### Chemicals and reagents

SQ109, bithionol, and ciprofloxacin (≥98% purity) were purchased from Sigma Aldrich (Sigma Aldrich, MO, USA). Phospholipids (≥99% purity) were purchased from Avanti Polar Lipids Inc. (Alabaster, AL, USA). All other chemicals and reagents were purchased from Fisher Scientific (MA, USA). The synthesis of the 14 SQ109 analogs was carried out as previously described and tested as monofumarate salts (26–28). A brief synthetic procedure is provided in the supplementary material (Fig. S1 and S2). The final yields of the ethylenediamine analogs ranged from 31% to 38%. All solvents and chemicals used for synthesis were purchased without further purification except Me_3_SiCl, which was distilled just before use. All synthesized compounds were purified by column chromatography and confirmed to have ≥95% purity by HPLC-MS analysis. SQ109, its 14 analogs, and bithionol were dissolved in DMSO; all other compounds were dissolved in ddH_2_O. Each compound was prepared at a stock concentration of 10 mg/mL.

### Bacterial strains and growth conditions

*Staphylococcus aureus* strains were grown on tryptic soy broth (TSB) (Becton Dickinson, NJ, USA). For overnight cultures, a single colony of each strain was inoculated into 5 mL of the appropriate medium and incubated at 37°C with shaking at 220 rpm for 18–24 h.

### Antimicrobial susceptibility assay

The MICs were determined by broth microdilution assays, following the guidelines of the Clinical and Laboratory Standards Institute (29). Briefly, overnight bacterial cultures were diluted to approximately 1 × 10^6^ CFU/mL in Mueller-Hinton broth 2, cation-adjusted (caMHB). Then, 50 μL from the diluted culture was added to the wells of 96-well plates (Corning, NY, USA) containing 50 μL of test compounds, pre-diluted in caMHB at twice the desired final concentration. A non-treated sample served as a negative control, and a sample containing only media was used as a blank. After an 18-h incubation at 37°C, MICs were determined by measuring the optical density at 600 nm (OD_600_) using a SpectraMAX 250 microplate reader (Molecular Devices, CA, USA) with bacterial growth defined as an OD_600_ reading of ≥0.05. Microdilution susceptibility assays were conducted in triplicate with at least two independent biological replicates.

### Time-kill assay

To generate exponential-phase cells, overnight cultures of *S. aureus* strains MW2 and VRS1 were diluted 1:10,000 into 25 mL TSB in a 250 mL flask and incubated at 37°C with shaking at 225 rpm for 4 h. Cells were washed three times with an equal volume of phosphate-buffered saline (PBS) and resuspended in the same buffer to an OD_600_ of approximately 0.4. Following this, 1 mL of the exponential-phase cell culture was mixed with 1 mL of PBS containing twice the desired concentration of compounds in a 96-well assay block (Corning, NY, USA). The assay block was sealed with a gas-permeable Breathe-Easy membrane and incubated at 37°C with shaking at 225 rpm. At specific time points, 200 μL of samples was removed, serially diluted 10-fold in PBS, and spot-plated onto tryptic soy agar plates. After 18 h of incubation at 37°C, bacterial colonies were counted. All experiments were conducted in triplicate.

Persister cells of *S. aureus* strains MW2 and VRS1 were prepared as previously described (30). In brief, cultures were grown for 18 h at 37°C with shaking at 225 rpm, followed by treatment with gentamicin at a final concentration of 20 μg/mL for 4 h. Cells were washed three times with PBS and resuspended to an OD_600_ of approximately 0.4. Then 1 mL of the persister suspension was transferred to a 96-well assay block (Corning, NY, USA) containing 1 mL of PBS with twice the desired concentration of the compound of interest. Ciprofloxacin and bithionol, a molecule known to kill stationary phase persisters (31), were used as an antibiotic and a positive control, respectively. Sampling, dilution, and plating were performed as outlined for exponential-phase cells.

### AMR development assay

To generate resistant mutant *S. aureus* strains, we followed a previously published procedure (32). Briefly, an overnight culture of MRSA-MW2 was resuspended in caMHB medium to an OD_600_ of approximately 0.003. From this diluted culture, 50 μL was placed into each well of a 96-plate containing 50 μL of an expanded gradient of drug concentrations. Ciprofloxacin was included as a positive control. The extended gradient was prepared by twofold serial dilutions, starting from initial concentrations of 40, 48, and 64 μg/mL for each compound, resulting in 24 distinct concentrations ranging from 0.156 to 32 μg/mL. After 24-h incubation at 37°C, OD_600_ was measured using the SpectraMAX 250 microplate reader, and bacterial growth was defined as an OD_600_ ≥ 0.05. Then, 4 μL from the well with growth at the highest drug concentration was diluted 1,000-fold in caMHB and transferred to a fresh gradient plate. This serial passage assay was conducted over 15 days using two independent cultures.

### DiSC_3_(5) membrane potential assay

To evaluate the bacterial membrane potential, we used the fluorescent dye DiSC_3_(5) (3,3′-dipropylthiadicarbocyanine iodide). *S. aureus* MW2 cells were grown to exponential phase in TSB from an overnight culture. The cells were washed thrice with PBS and then adjusted to an OD_600_ of 0.1. To energize the bacteria, we added 25 mM glucose and incubated the suspension at 37°C with shaking for 15 min. DiSC_3_(5) was then added to a final concentration of 1 μM, and the mixture was kept in the dark for 10 min. Next, 90 μL of the bacterial suspension was transferred into the wells of a black, clear-bottom 96-well plate (Corning, NY, USA), and fluorescence was monitored for 30 min using a Cytation 5 multimode reader (BioTek, VT, USA), with excitation at 620 nm and emission at 675 nm. Subsequently, 10 μL οf serially diluted compound or PBS solution was added to the wells of the 96-well plate, and fluorescence was recorded every 3 min for 1 h at room temperature.

### SYTOX membrane permeability assay

The SYTOX green membrane permeability assay was conducted following a published protocol (33). Briefly, *S. aureus* MW2 exponential or persister cells were washed thrice in PBS and resuspended to an OD_600_ of 0.4. SYTOX Green (Invitrogen, CA, USA) was then added to the diluted cell suspension at a final concentration of 5 μM, and the samples were incubated in the dark at room temperature for 30 min. Next, 50 μL of the dye/bacteria mixture was added to each well of a black, clear-bottom 96-well plate containing 50 μL of serially diluted compound at 2× the final concentration. Fluorescence was monitored every 5 min for 2 h at room temperature using a Cytation 3 multimode plate reader (BioTek, VT, USA) at an excitation wavelength of 485 nm and an emission wavelength of 528 nm.

### Propidium iodide permeability assay

For the propidium iodide (PI) membrane permeability assays, we followed a similar procedure to that of the SYTOX green dye. Briefly, we washed exponential-phase *S. aureus* MW2 cells three times and adjusted them to an OD_600_ equal to 0.4 in PBS. We then added PI (Invitrogen, CA, USA) to a final concentration of 2 μM and left it to incubate for 15 min in the dark at room temperature. The bacteria/dye suspension was mixed with the compound in a 1:1 ratio, and the fluorescence was recorded using the Cytation 5 multimode reader set at excitation and emission wavelengths of 540 and 620 nm, respectively.

### ATP leakage assay

Leakage of intracellular ATP from exponential-phase and persister *S. aureus* MW2 cells was evaluated based on a previously described protocol with some modifications (34). In short, the cells were washed three times with PBS and adjusted to an OD_600_ of approximately 0.4. Next, 200 μL of the bacterial suspension was mixed with 200 μL of the compound at 2× the desired final concentration in a 1.5 mL conical tube. The tubes were incubated at 37°C for 1 h with shaking. Following incubation, the samples were centrifuged at 14,000 × *g* for 5 min. Then, 50 μL from the supernatant fraction of each tube was transferred to the wells of a white 96-well plate (Greiner Bio-One, NC, USA) and mixed with 50 μL of BacTiter-Glo reagent (Promega, WI, USA). After a 5-min incubation in the dark at room temperature, luminescence was measured using the Cytation 5 multimode reader.

### Scanning electron microscopy

Scanning electron microscopy (SEM) was conducted according to an existing protocol with minor modifications (35). *S. aureus* MW2 cells from an overnight culture were diluted 1:1,000 and incubated at 37°C with shaking for 3 h. The bacterial culture was washed three times with PBS and resuspended to an OD_₆₀₀_ ≈ 0.4. A 1 mL aliquot of the bacterial suspension was transferred to a microcentrifuge tube containing 1 mL of compound at a final concentration of 40 μg/mL in PBS. The tubes were incubated at 37°C for 1 h, followed by centrifugation at 14,000 × *g* for 5 min. The resulting cell pellet was washed twice with PBS and fixed overnight at 4°C in 2.5% glutaraldehyde. Next, 30 μL of the fixed cell suspension was applied onto a (3-aminopropyl) triethoxysilane (APTES)-functionalized Si (100) wafer and incubated for 1 h at room temperature. The wafer was washed three times with PBS, followed by progressive dehydration with ethanol solutions in ascending concentrations (30%, 50%, 70%, 90%, and 100% vol/vol in water), each for 15 min. Subsequently, 30 μL of tert-butyl alcohol (50% vol/vol in ethanol) was applied, and samples were allowed to air-dry. The wafers were mounted on aluminum SEM sample holders and coated with a thin Pt/Pd film (7 nm) using a Magnetron 208HR High-Resolution Sputter Coater (Ted Pella Inc., CA, USA). Samples were visualized at room temperature under high vacuum using a Nova NanoSEM 230 (FEI, OR, USA), with a working distance of 5 mm and an accelerating voltage of 5 kV.

### Molecular dynamics simulations

Molecular structures of SQ109 and its analogs were first sketched in Marvin Sketch software (v. 24.1.2, 2024, ChemAxon) and then imported into the Maestro interface (36). We subsequently calculated physicochemical properties, including topological polar surface area (TPSA; the sum of the surface areas of all polar atoms, such as nitrogen and attached hydrogen atoms) and lipophilicity (cLogP), for each compound (Table 1). The ligands underwent preparation using the ligand preparation LigPrep (36) module in Maestro. Protonation was performed utilizing Epik (37), at pH 7, and the conformation energy of the ligands was minimized with the OPLS 2005 force field (38). Additionally, versions of the ligands were generated without any ionization states (charges).

**TABLE 1:** Antibacterial activity of SQ109 and its analogs against MRSA pathogens^*b*^

Comp. no	Chemical structures	MW	cLogP	TPSA	MIC^*a*^
		(g/mol)		(Å^2^)	(μg/mL)
SQ109	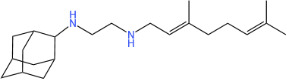	330.6	4.11	24.06	16
AK108	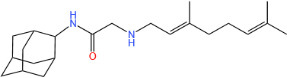	344.5	5.07	41.13	64
AK112	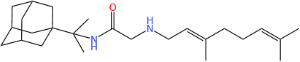	386.6	4.95	41.13	8
AK116	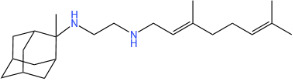	344.6	4.17	24.06	32
AK117	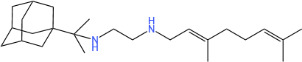	372.6	5.20	24.06	8
AK118	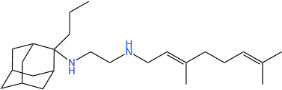	372.6	6.22	24.06	8
AK119	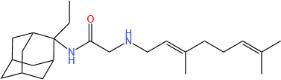	372.6	5.12	41.13	64
AK120	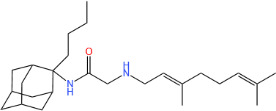	400.6	5.35	41.13	8-16
AK121	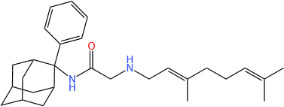	420.6	4.29	41.13	32
AK122	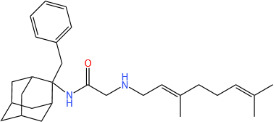	434.7	5.35	41.13	64
AK123	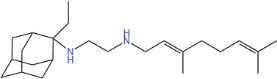	358.6	6.08	24.06	8-16
AK125	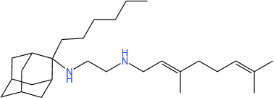	414.7	6.18	24.06	16
AK126	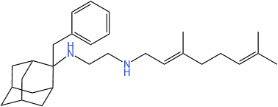	420.7	5.43	24.06	2
AK127	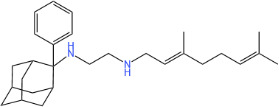	406.7	5.47	24.06	4
Vanc.		N.C.	N.C.	N.C.	0.5
Gent.		N.C.	N.C.	N.C.	1

^*a*^ Measured against *S. aureus* MW2.

^*b*^ Gent., gentamicin; MW, molecular weight; TPSA, topological polar surface area; Vanc., vancomycin; N.C., not calculated.

To model the MRSA lipid bilayer, we used a model that has been previously published (33). This membrane model consists of dioleoyl-*sn*-glycero-3-phosphocholine (DOPC) and 1,2-dioleoyl-*sn*-glycero-3-phospho- (1′-*rac*-glycerol) (DOPG) at 7 to 3 ratio. To generate the lipid bilayer and the simulation box, we used the Packmol-memgen (39, 40) module within Amber24 (41). This module uses Packmol as a packing engine to place the simulation components within the box. Specifically, to generate the membrane system, we used DOPC and DOPG lipids with 7:3 ratios accordingly. Additionally, we introduced NaCl at a concentration of 0.15 M. The systems generated by Packmol consist of approximately 95,000 atoms, which consist of 32,500 (242 residues) lipids and 62,500 (21,000 residues) waters. The final membrane simulation box has dimensions of 111 × 106 × 119 Å^3^. To add parameters to the membrane system, the Leap module was utilized (41). We applied the lipid21 (42) force field and TIP3P (43) water parameters to model all lipid and water interactions.

After the generation and parametrization of the bilayer, the system was subjected to an energy minimization phase, which consisted of 2,500 steps utilizing the steepest descent algorithm (44) and another 2,500 steps using the conjugate gradient method (45). The cutoff of non-bonded interactions was set to 12 Å for all simulations, and positional restraints of 5 kcal mol^−1^ Å^−2^ to the lipid heads were implemented. After completing the minimization process, we subjected the system to two consecutive NVT steps, followed by four NPγT steps. During these phases, we implemented a reduction in position restraints to maintain membrane stability and ensure optimal lipid packing. To control the temperature and volume, we used the Langevin Dynamics (46) (T = 310K) with a constant friction coefficient at 1 ps^−1^ (47). To control the pressure in all NPγT steps, we applied the Berendsen barostat (48) using semi-isotropic conditions, an external target pressure of 1 bar, and 2 ps pressure relaxation time. The SHAKE algorithm (47) was enabled for all calculations performed. The lipid positional restraints were 5 kcal mol^−1^ Å^−2^, 2.5 kcal mol^−1^ Å^−2^, 1 kcal mol^−1^ Å^−2^, 0.5 kcal mol^−1^ Å^−2^, 0.1 kcal mol^−1^ Å^−2^, and 0.01 kcal mol^−1^ Å^−2^ for each of the six equilibration steps accordingly. Each NVT step was 125 ps, which was the first NPγT step. The rest of the equilibration NPγT steps were 500 ps each. Following the equilibration phase, we performed an unrestrained NPγT production phase for 500 ns, writing the coordinates every 500 picoseconds, having a total of 5,000 frames in the MD simulations. The energy minimization step was performed and calculated using the central processing unit of the workstations by implementing the pmemd algorithm. The rest of the equilibration steps, including the unrestrained production, were calculated and run via the pmemd algorithm, but initializing GPU support (pmemd.CUDA) (49, 50).

Upon extracting the last frame of the MD simulation of the membrane-only system, we used the parmed (51) module to convert the amber topology file to a (52) topology file. Each ligand molecule was parametrized using the GAFF2 force field (53, 54) by means of the acpype module (55), and ligand-lipid interaction was calculated with the GAFF2 force field (53). Then, we utilized the Gromacs insert-molecules command to add one ligand molecule within the solvation right above the lipid heads of the membrane. The membrane-ligand system was subjected to a very short equilibration of 250 ps using v-rescale and c-rescale thermostat (56) and barostat (57), at 310K and pressure 1 bar, respectively. Lastly, the system was subjected to a production phase of 500 ns using the LINCS constraints algorithm (58). Each membrane-ligand complex was subjected to two replica MD with randomized velocities.

In each molecular trajectory, we used trjconv via Gromacs to center all lipid atoms and then view the trajectory using visual molecular dynamics (VMD) (59). Further analysis was performed using Gromacs density and hydrogen bond commands.

### Synergy testing

The checkerboard microdilution assay was used to assess the interaction between SQ109 and conventional antibiotics (60). Briefly, twofold serial dilutions of SQ109 were combined with twofold serial dilutions of each antibiotic in a 96-well plate to generate an 8 × 8 concentration matrix. An overnight culture of MRSA MW2 was diluted in cation-adjusted Mueller-Hinton broth (caMHB) to a final inoculum of 1 × 10⁶ CFU/mL, and 50 μL of the bacterial suspension was added to each well, resulting in a final volume of 100 μL. Plates were incubated at 37°C for 18 h, and bacterial growth was measured at OD₆₀₀ using a SpectraMAX 250 microplate reader. The fractional inhibitory concentration index (FICi) was calculated as:


$$FICi=\left(MIC_{ab}/MIC_{a}\right)+\left(MIC_{ba}/MIC_{b}\right)$$

where MIC_a_ and MIC_b_ represent the MIC values of compounds A and B alone, and MIC_ab_ and MIC_ba_ represent the MICs of compounds A and B, respectively, in combination. Interactions were interpreted as follows: FICi ≤ 0.5, synergy; 0.5 < FICi ≤ 4, no interaction; and FICi > 4, antagonism (61).

To evaluate synergy in MRSA MW2 persister cells, we used a time-kill assay format as described in section Time-Kill Assay. Synergy was defined as a ≥2 Log10 decrease in CFU/mL between the combination and the most active constituent alone (60).

### Biofilm inhibition assay

The inhibition of biofilm by the SQ109 analogs was assessed using a previously described protocol with modifications (62). In brief, overnight cultures from *S. aureus* strains MW2 and VRS1 were resuspended in TSB supplemented with 3% NaCl and 0.2% glucose (final OD_600_≈0.02). Next, 50 μL of each bacterial solution was added to the wells of a 96-well plate containing 50 μL of compound at 2× the desired concentration, or 50 μL of medium as a negative control. Plates were incubated at 37°C under static conditions for 24 h. After incubation, wells were gently washed with PBS (Fisher Scientific, MA, USA) to remove non-adherent cells. Biofilm viability was assessed by adding 100 μL of TSB containing 20% CyQUANT XTT (Fisher Scientific, MA, USA) to each well, followed by a 2-h incubation at 37°C. The absorbance was measured at 450 nm using the SpectraMax 250 plate reader, and the percentage of viable cells was calculated by comparing the treated wells to the untreated bacterial control.

### Biofilm disruption assay

The effects of SQ109 and its analogs were evaluated using a previously described biofilm disruption assay with minor modifications (30). To allow biofilm formation, 90 μL of bacterial suspension (OD_600_≈0.01) in TSB supplemented with 3% NaCl and 0.2% glucose was added to the wells of a 96-well plate and left to incubate at 37°C under static conditions for 24 h. Subsequently, the wells were gently washed with PBS to remove loosely attached cells, and 100 μL of serially diluted compounds (32–1 μg/mL) prepared in the same medium was added to the wells, and the plates were incubated under the same conditions for another 24 h. After the second incubation, non-adherent cells were removed by carefully washing the wells with PBS (Fisher Scientific, MA, USA). Biofilm viability was then determined by adding 100 μL of TSB containing 20% CyQUANT XTT (Fisher Scientific, MA, USA) to each well. Plates were incubated for 2 h at 37°C, after which absorbance was measured at 450 nm using the SpectraMax 250 plate reader. The percentage of viable cells in biofilm was calculated by comparing the treated wells with those of the untreated bacterial control.

### Fluorescence microscopy of mature biofilms

An overnight culture of *S. aureus* MW2 was resuspended to a final OD_600_ ≈ 0.01 in fresh TSB supplemented with 3% NaCl and 0.2% glucose. Biofilms were formed by adding 500 μL of bacterial suspension to the chambers of Lab-Tek Chambered coverglass (Cat. No. 155383, Thermo Fisher Scientific, USA) and maintaining them at 37°C for 24 h under static conditions. After incubation, we carefully washed the wells with PBS (Fisher Scientific, MA, USA), treated the established biofilm with 500 μL of the analogs in fresh medium, and left it to incubate at 37°C for an additional 18 h. The slides were washed again with PBS to remove any loosely attached cells, and the biofilms were stained with 50 μL of staining reagent using the LIVE/DEAD BacLight Bacterial Viability Kit (Invitrogen, CA, USA) according to the manufacturer’s instructions. We visualized the biofilms using the Olympus Fluoview FV3000 confocal microscope (Olympus, Tokyo, Japan).

### Hemolytic and cytotoxic assays

The hemolytic activity of compounds was performed as previously described (33). Human red blood cells (hRBCs; 10%) were purchased from Rockland Immunochemicals (Limerick, PA, USA). The hRBCs were washed three times with PBS and resuspended to a final concentration of 4%. Then 100 μL of the cell suspension was added to each well of a 96-well plate containing 100 μL of twofold serially diluted compounds in PBS. DMSO at a final concentration of 1.28% served as a negative control, while 1% Triton X-100 was used as a positive control. The plate was incubated at 37°C for 1 h, followed by centrifugation at 1,300 × *g* for 10 min. Finally, 100 μL of the supernatant fraction was transferred to a new 96-well plate, and absorbance was measured at 540 nm. The percentage of hemolysis was calculated using the following equation:


$$Hemolysis\%=\left[\left(Abs_{540\left(sample\right)}-Abs_{540(DMSO)}\right)/\left(Abs_{540(1\%TritonX-100)}-Abs_{540(DMSO)}\right)\right]\times 100\%$$

The cytotoxicity of the compounds was assessed using the CyQUANT XTT cell viability assay kit (Fisher Scientific, MA, USA) in two cell lines: HepG2 (human hepatocellular carcinoma) and HKC-8 (human renal proximal tubular) cells. Cells were cultured at 37°C with 5% CO_2_ in Dulbecco’s Modified Eagle Medium (DMEM) supplemented with 10% heat-inactivated fetal bovine serum and 1% penicillin/streptomycin. At approximately 85% confluence, cells were harvested and seeded in a 96-well plate at a density of 5 × 10^5^ cells/well for 24 h at 37°C with 5% CO_2_. Cells were subsequently treated with 100 μL twofold serially diluted compound in DMEM for an additional 24 h. After treatment, 70 μL of the XTT-electron coupling reagent mixture was added to each well and incubated for 4 h. Absorbance was measured at 450 nm and 660 nm using the SpectraMax 250 plate reader. Vancomycin was included as an antibiotic control, 1% Triton X-100 as a positive control, and 0.64% of DMSO as a negative control; wells with medium only were used as blanks. Cell viability was calculated according to the manufacturer’s instructions.

### Liposomal synthesis

DMPC liposomes were prepared with incorporated AK126 or AK127 at 10% wt/wt of compound per phospholipid. This was achieved by mixing different amounts of CHCl_3_:MeOH 9:1 vol/vol stock solutions, depending on the solubility of each molecule (DMPC 10 mg/mL, AK126 1 mg/mL, and AK127 0.9 mg/mL), preparing lipid films through evaporation, and subsequently hydrating the films with aqueous media. Briefly, the mixtures were transferred into round flasks and connected to a rotary evaporator (Rotavapor R-114, Buchi, Switzerland), a vacuum of −1 atm was applied, and lipid films were formed at 40°C while stirring. The mixed films were then stored for 24 h and were then hydrated with a sucrose solution 286 μM (286 mOsm), by slowly stirring for 1 h in a water bath, above the phase transition temperature of the lipid (40°C for DMPC), for a total phospholipid concentration of 10 mg/mL. The resultant milky suspensions were then subjected to two 5-min sonication cycles (amplitude 70%, cycle 0.5 s) interrupted by a 5-min resting period, using a probe sonicator (UP 200S, Dr. Hielsher GmbH, Berlin, Germany). The resultant clear suspensions were allowed to anneal for 30 min before they were measured for their physicochemical properties. The mean particle size (hydrodynamic diameter, Dh), size distribution (polydispersity index, PDI), and surface charge (zeta potential, ζ-pot) of the obtained nanoparticles were investigated by dynamic and electrophoretic light scattering (DLS and ELS), by utilizing a photon correlation spectrometer (Zetasizer 3000 HSA, Malvern, UK) (Table S1). The samples were prepared by diluting aliquots of the suspensions 30-fold in HPLC-grade H_2_O. Measurements were performed in triplicate at 25°C at a detection angle of 90° and analyzed by the CONTIN method (MALVERN software). Finally, the physical/colloidal stability of the nanoparticles was assessed during storage at 4°C by measuring their size and polydispersity.

### *C. elegans* infection model

The *C. elegans*-MRSA infection assay was performed as previously outlined with minor modifications (63). Approximately 3,000 synchronized L1-stage larvae of the temperature-sensitive, sterile, and immunocompromised *C. elegans* strain AU37 [*glp-4(bn2);sek-1(km4*)] were dispensed onto an *E. coli* HB101 lawn on 10-cm slow-kill agar plates. After incubation at 25°C for approximately 52 h, the worms were washed six times with 40 mL of M9 buffer and resuspended at a concentration of 1,000 worms/mL. For the *S. aureus* infection, MW2 cells were grown overnight and diluted in M9 buffer supplemented with 20% TSB to achieve a final OD_₆₀₀_ of 0.08.

The infection assay was set up on a half-area 96-well plate (Corning, NY, USA). In each well, we added 20 μL of M9 buffer containing the desired concentration of the test compound or vancomycin (positive control), followed by 15 μL of worm suspension (20–25 worms), and 35 μL of the diluted *S. aureus* MW2 suspension. The plates were sealed with an air-permeable membrane and incubated at 25°C for 5 days. After incubation, the worms were washed nine times with M9 buffer using a 405LS microplate washer (BioTek, VT, USA). To assess viability, the worms were stained overnight with 50 μL/well of 1 μM SYTOX Orange (Invitrogen, CA, USA). After removal of the excess strains by washing an additional three times, fluorescence and bright-field images were captured using the Cytation 3 multimode reader.

### Data analysis

All experiments were performed with a minimum of three independent biological replicates, unless otherwise specified. Each data point in the figures denotes the mean value, with error bars indicating the standard deviation (SD). Statistical differences between non-treated and compound-treated groups were assessed using one-way ANOVA followed by Dunnett’s post hoc test (GraphPad Prism, version 10.1.2). Significant thresholds were defined as follows: *P < 0.05, **P < 0.01, ***P < 0.001 and ****P < 0.0001.

## RESULTS

### Antibacterial susceptibility of SQ109 and its analogs against MRSA

We initially tested the efficacy of SQ109 and its 14 analogs against MRSA (Table 1). In our hands, SQ109 exhibited moderate inhibitory activity (MIC = 16 µg/mL). Compared to SQ109, analogs AK112, AK117, AK118, AK120, AK123, AK125, AK126, AK127, and AK133 showed similar or improved antimicrobial activity against MRSA, with AK126 being the most potent (MIC = 2 µg/mL), closely followed by AK127 (MIC = 4 µg/mL). In contrast, analogs AK108, AK116, AK119, AK121, and AK122 exhibited weaker activity than SQ109.

To further confirm the antimicrobial activity of SQ109 and its two most potent ethylenediamine analogs—AK126 and AK127—we used a panel of MDR *S. aureus* clinical isolates (7) as well as the VanA-type vancomycin-resistant strain VRS1 (64). Both SQ109 and analogs AK126 and AK127 maintained relatively consistent MICs of 16, 2, and 4 μg/mL, respectively (Table 2).

**TABLE 2:** Minimum inhibitory concentrations of SQ109 and analogs AK126 and AK127 against a panel of *S. aureus* strains

	MIC (μg/mL)			
*S. aureus* strain	SQ109	ΑΚ126	ΑΚ127	Vanc.^*a*^
Newman	16	2	4	0.5
USA300	16	2	4	1
BF2	16	2	4	1
BF3	16	2	4	1
BF4	16	4	8	1
BF5	32	2	4	0.5
BF6	16	2	4	0.5
BF7	32	2	4	1
BF8	32	2	4	0.5
BF9	16	2	4	0.5
BF10	16	2	4	1
BF11	16	4	8	1
VRS1	16	2	4	>64

^*a*^ Vanc., vancomycin.

**Fig 1:**
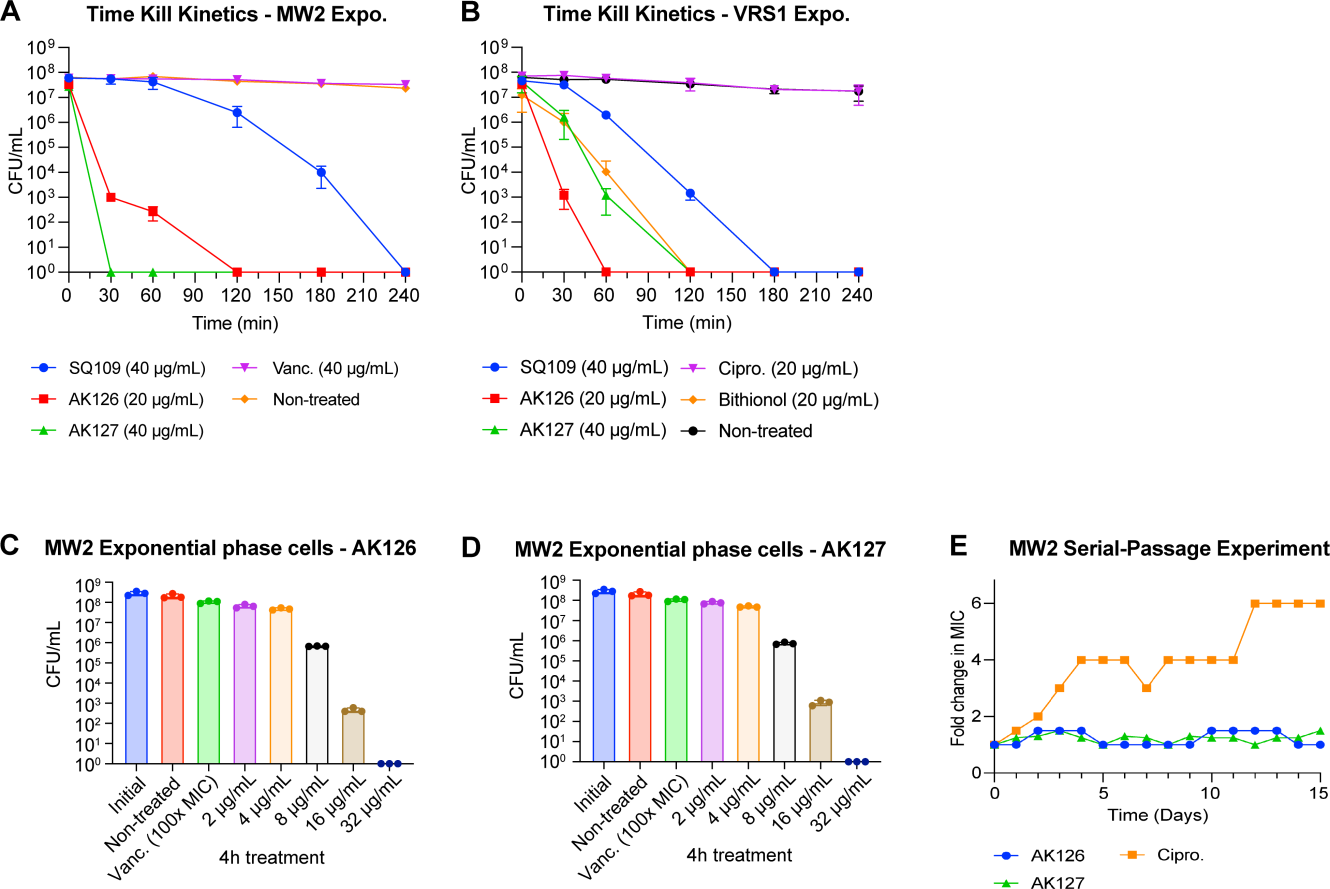
Bactericidal activity and resistance profile of SQ109 analogs against *S. aureus*. (**A, B**) Time-kill kinetics of AK126 and AK127 against exponential-phase *S. aureus* MW2 (**A**) and VRS1 cells (**B**) at 10× MIC. The parent compound, SQ109 (at 40 μg/mL), and a non-treated group were included as reference and bacterial control, respectively (*n* = 3, replicated thrice). Vancomycin (at 40 μg/mL) and ciprofloxacin (at 20 μg/mL) were used as antibiotic controls against *S. aureus* MW2 and VRS1, respectively. Bithionol (at 20 μg/mL) was included as a positive control for experiments with VRS1 cells. (**C, D**) Dose-dependent killing of MRSA-MW2 exponential-phase cells after 4-h treatment with various concentrations of AK126 (2–32 μg/mL) (**C**) or AK127 (2–32 μg/mL) (**D**) (*n* = 3, replicated thrice). (**E**) *S. aureus* MW2 resistance development assay (*n* = 2, replicated twice).

To determine the bactericidal or bacteriostatic properties of analogs AK126 and AK127**,** we performed time-kill assays using exponential-phase MRSA-MW2 cells. At 10× MIC, both compounds demonstrated rapid bactericidal activity, reducing approximately 3 × 10⁷ CFU/mL below the limit of detection within 2 h for AK126 and 30 min for analog AK127 (Fig. 1A). These killing rates surpassed those observed for SQ109 (at 40 μg/mL) and vancomycin (at 40 μg/mL). Similar results were obtained against exponential-phase VRS1 cells (Fig. 1B), both analogs eradicated approximately 5 × 10^7^ CFU/mL within 2 h at 10× MIC. These results were comparable to the membrane-targeting agent bithionol, which also eradicated all VRS1 cells within 2 h (at 20 μg/mL). In contrast, ciprofloxacin (at 20 μg/mL) failed to reduce the number of CFU/mL over 4 h.

In addition, we determined the dose–response relationship of AK126 and AK127 against exponential-phase MRSA-MW2 cells after 4 h of treatment (Fig. 1C and D). At concentrations below 8 μg/mL, neither compound reduced CFU/mL. At 8 μg/mL, both analogs reduced CFU/mL by ~3 Log_10_, and at 16 μg/mL, the reduction reached ~6 Log_10_. At 32 μg/mL, both analogs reduced bacterial counts from an initial ~2 × 10⁸ CFU/mL to below the limit of detection.

### Absence of detectable resistance development

We evaluated whether continuous exposure to AK126 or AK127 could induce resistance in *S. aureus*. To do this, we subcultured MRSA-MW2 cells for 15 days in sub-MIC concentrations of each analog, and we included ciprofloxacin as a positive control (Fig. 1E). Ciprofloxacin exposure led to a twofold increase in the MIC of MRSA-MW2 by day 5 and a sixfold increase by day 15. However, exposure to AK126 and AK127 did not significantly alter the MIC after 15 days, indicating a reduced likelihood of resistance development for these analogs.

### SQ109 and its analogs compromise membrane integrity in growing MRSA cells

**Fig 2:**
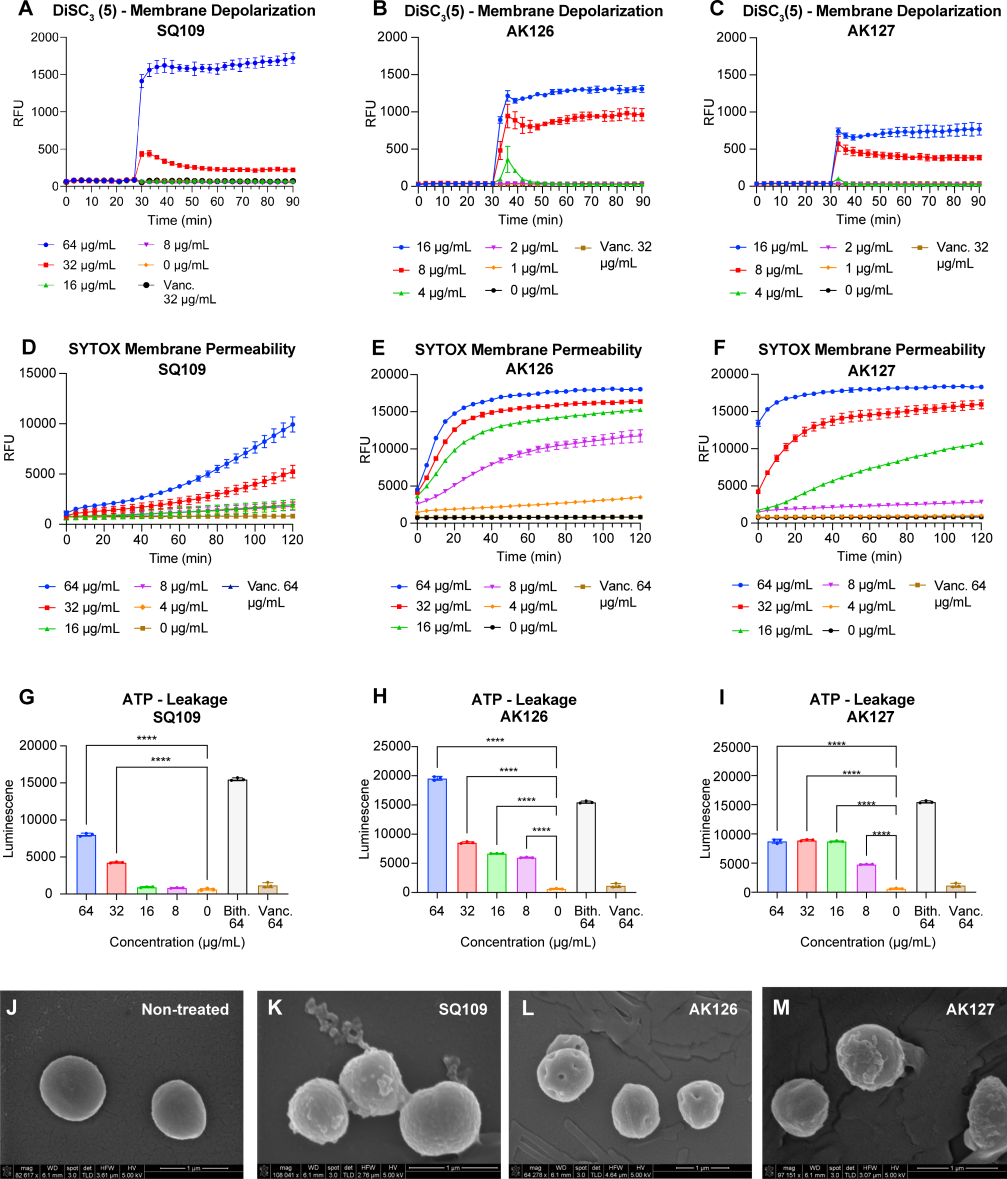
SQ109, AK126, and AK127 disrupt the membrane lipid bilayer on *S. aureus* cell. (**A–C**) Uptake of DiSC_3_(5) dye after the exposure of exponential-phase *S. aureus* MW2 cells at 30 min with varying concentrations of SQ109 (**A**), AK126 (**B**), and AK127 (**C**), and monitored for 1 h (*n* = 3, replicated thrice). (**D–F**) Uptake of SYTOX green dye after the exposure of *S. aureus* exponential-phase MW2 cells with varying concentrations of SQ109 (**D**), AK126 (**E**), and AK127 (**F**) monitored for 2 h (*n* = 3, replicated thrice). (**G–I**) ATP-leakage from exponential-phase *S. aureus* MW2 cells after treatment with SQ109 (**G**), AK126 (**H**), and AK127 (**I**) at varying concentrations for 1 h (*n* = 3, replicated twice, ****P < 0.0001 by one-way ANOVA followed by Dunnett’s multiple comparison test). (**J–M**) SEM images of exponential-phase *S. aureus* MW2 (**J**) and after treatment with 40 μg/mL of SQ109 (**K**), AK126 (**L**), and AK127 (**M**) for 1 h (replicated twice).

Given the rapid bactericidal activity of analogs AK126 and AK127 and their low propensity for resistance development, we investigated their effects on the PMF and the membrane in exponential-phase MRSA-MW2 cells. To assess their impact on the PMF, we employed a DiSC_3_(5) fluorescence-based dye assay. Both AK126 and AK127 induced a dose-dependent increase in fluorescence instantly after exposure of cells at concentrations as low as 4 and 8 μg/mL (2× MIC), respectively (Fig. 2B and C), indicating membrane depolarization and PMF disruption. Likewise, SQ109 triggered an increase in DISC_3_(5) fluorescence almost immediately after exposure of cells with 32 μg/mL (2× MIC) or 64 μg/mL (4× MIC) (Fig. 2A).

Next, we examined whether treatment with AK126 and AK127 affects the integrity of the membrane using two membrane-impermeable DNA-binding fluorescent dyes, SYTOX green and PI. After exposing exponential-phase MRSA-MW2 cells to AK126 and AK127, we observed a rapid, dose-dependent increase in fluorescence with both SYTOX green (Fig. 2E and F) and PI (Fig. S3B and C), indicative of membrane disruption. SQ109 showed a similar effect, increasing fluorescence at concentrations equal to or exceeding 2× MIC. The results were consistent with both the SYTOX green (Fig. 2D) and the PI dye (Fig. S3A). In contrast, vancomycin, which does not cause membrane damage, did not produce any increase in fluorescence.

**Fig 3:**
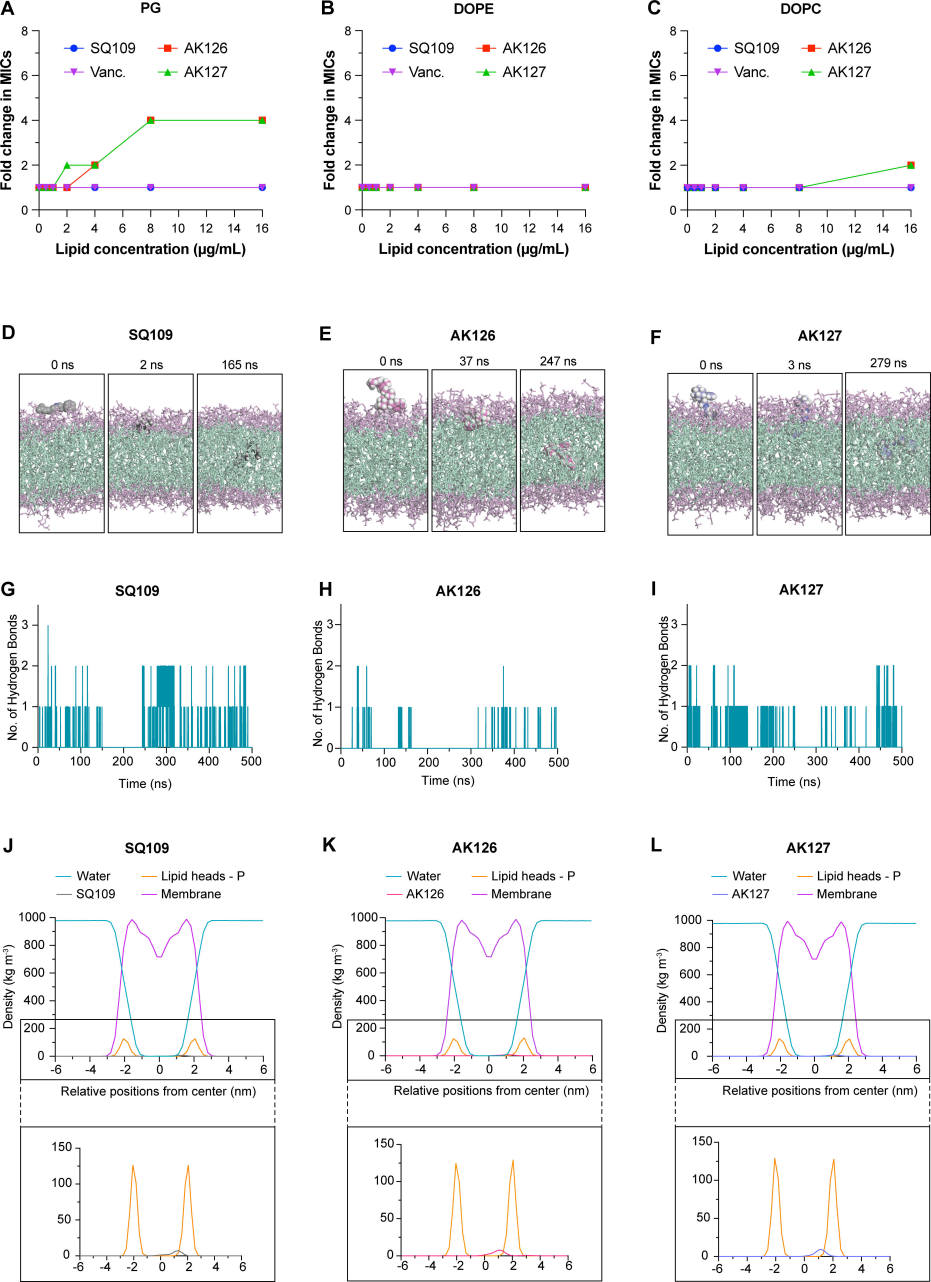
AK126 and AK127 preferentially interact with anionic phospholipids and display strong binding to gram-positive membranes. (**A–C**) MICs of SQ109, AK126, and AK127 in the presence of PG, phosphatidylethanolamine (DOPE), or phosphatidylcholine (DOPC). Concentrations ranged from 0 to 16 μg/mL. Vancomycin was used as a control. (**D–F**) Snapshots showing SQ109 (**D**), AK126 (**E**), and AK127 (**F**) at the beginning of the MD simulation (left), at the moment of the initial attachment (center), and their lowest position within the bilayer (right). (**G–I**) Number of hydrogen bonds of SQ109 (**G**), AK126 (**H**), and AK127 (**I**) with the membrane. (**J–L**) Density profiles of SQ109 (**J**), AK126 (**K**), and AK127 (**L**). The MD simulations were conducted in two independent replicates. The provided snapshots and plots illustrate one representative replicate.

To further assess the impact of AK126 and AK127 on the bacterial membrane, we performed a luciferin-luciferase bioluminescence assay to investigate whether they induce leakage of intracellular ATP. After the exposure of exponential-phase MRSA-MW2 cells to AK126 or AK127 for 1 h, we noted a significant luminescence increase (*P* < 0.0001) at concentrations ≥8 µg/mL for both analogs (Fig. 2H and I). In comparison, SQ109 produced similar results at concentration ≥32 µg/mL, whereas vancomycin did not cause any increase in luminescence at 64 μg/mL (Fig. 2G).

We also examined morphological changes induced by SQ109, AK126, and AK127 using SEM. Compared with untreated MRSA-MW2 cells (Fig. 2J), cells exposed to SQ109 (40 µg/mL) for 1 h induced a heterogeneous, rough membrane surface (Fig. 2K). Notably, treating cells with AK126 at 40 μg/mL resulted in distinct membrane invaginations (Fig. 2L), absent in cells exposed to the other compounds and the untreated cells. In comparison, cells treated with AK127 exhibited a heterogeneous rough membrane surface and pronounced blebbing (Fig. 2M), similar to the effects of SQ109. Taken together, these findings indicate that SQ109, AK126, and AK127 act as membrane-disrupting agents, ultimately leading to the death of *S. aureus* cells.

### SQ109 analogs preferentially interact with anionic phospholipid and penetrate the lipid bilayer

To gain additional insight into how SQ109 and its analogs interact with the MRSA membrane, we examined whether exogenous phospholipids could influence their antimicrobial activity. We selected PG, the major anionic phospholipid in staphylococcal membranes, along with phosphatidylethanolamine (PE; tested as DOPE) and phosphatidylcholine (PC; tested as DOPC). Interestingly, the presence of PG affected the MICs of both AK126 and AK127 in a dose-dependent manner, beginning at concentrations as low as 4 and 2 μg/mL, respectively, whereas the activity of SQ109 remained unaffected (Fig. 3A). Vancomycin, which was included as a control, showed no change in the MIC. In contrast, DOPE at concentrations up to 16 µg/mL did not alter the activity of either analog (Fig. 3B), and DOPC caused only a modest onefold increase in MICs at 16 µg/mL (Fig. 3C).

To further investigate how SQ109, AK126, and AK127 interact with the bacterial membrane at the molecular level, we performed a 500-ns, all-atom, molecular dynamics (MD) simulations using a model of a negatively charged lipid bilayer model (DOPC/DOPG 7:3). The MD simulations revealed that all three compounds initially bind to the membrane through hydrogen bonding between the ethylenediamine groups and the headgroups of the lipid bilayer. Among them, SQ109 was the first to bind, attaching to the membrane at 2 ns (Fig. 3D), followed by AK127 at 3 ns (Fig. 3F) and AK126 at 37 ns (Fig. 3E). Shortly after binding, all three molecules were able to penetrate the lipid bilayer through hydrophobic interactions between geranyl groups and the lipid tails. Despite binding first, SQ109 failed to form equally strong hydrophobic interactions and maintained one to two hydrogen bonds with the lipid headgroups throughout the simulation (Fig. 3G). In contrast, AK126 molecules formed stronger hydrophobic interactions after initial penetration, rarely forming hydrogen bonds with the lipid headgroups (Fig. 3H). AK127 exhibited an intermediate behavior, forming some hydrogen bonds with lipid head groups throughout the simulation (Fig. 3I). Density plot analysis further supports these observations. Over the 500-ns simulation, AK126 was positioned closest to the membrane center with an average density peak at 9.69 Å (Fig. 3K; Table S2), followed by AK127 at 10.93 Å (Fig. 3L; Table S2) and SQ109 at 12.16 Å (Fig. 3J; Table S2), indicating that AK126 achieved the deepest membrane penetration.

### Antimicrobial potency of SQ109 and its analogs against persister cells

**Fig 4:**
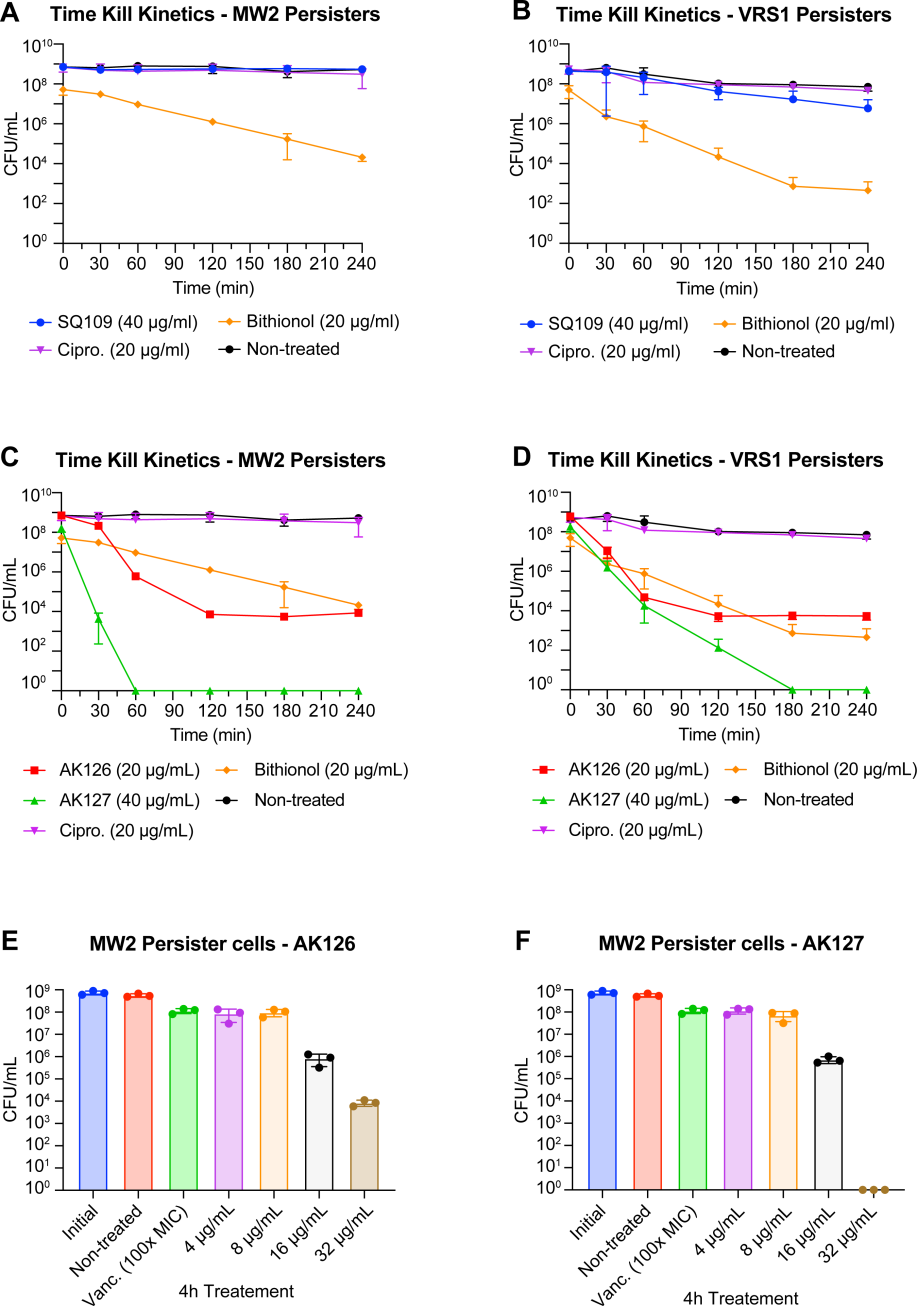
Activity of SQ109, AK126, and AK127 against gentamicin-induced *S. aureus* persister cells. (**A, B**) Time-kill kinetics of SQ109 (at 40 μg/mL) against gentamicin-induced *S. aureus* MW2 (**A**) and VRS1 (**B**) persister cells. (**C, D**) Time-kill kinetics of AK126 (at 20 μg/mL, 10× MIC) and AK127 (at 40 μg/mL, 10× MIC) against gentamicin-induced *S. aureus* MW2 (**C**) and VRS1 (**D**) persister cells. Ciprofloxacin (20 μg/mL) was used as an antibiotic control and bithionol (20 μg/mL) as a positive control (*n* = 3, replicated thrice). (**E, F**) Dose-dependent killing of MRSA-MW2 persister cells after 4-h treatment with various concentrations of AK126 (4–32 μg/mL) (**E**) or AK127 (4–32 μg/mL) (**F**) (replicated thrice).

To further assess the bactericidal activity of SQ109, AK126, and AK127, we tested their efficacy against persister cells from strains MW2 and VRS1. Treatment with SQ109 at 40 μg/mL showed limited activity, failing to reduce MRSA-MW2 persister cells (Fig. 4A) and lowering VRSA-VRS1 persister cells by only 1.8 Log_10_ within 4 h (Fig. 4B). In contrast, AK126 at 20 μg/mL (10× MIC) lowered the initial ~10^9^ CFU/mL by almost 5 Log_10_ within 2 h in both strains, while AK127 at 40 μg/mL (10× MIC) reduced the initial ~10^8^ CFU/mL below the limit of detection within 1 h for MRSA-MW2 persisters and within 3 h for VRSA-VRS1 persisters (Fig. 4C and D). By comparison, bithionol at 20 μg/mL (20× MIC), which has been shown to kill MRSA persisters (31), decreased MRSA persisters by 3.4 Log_10_ within 4 h and VRSA-VRS1 persister cells by 5 Log_10_ in 3 h. Ciprofloxacin at 20 μg/mL, included as an antibiotic control, did not affect either MRSA-MW2 or VRSA-VRS1 persister cells. To define the concentration–response relationship, we next examined persister killing at lower concentrations of AK126 and AK127 after 4 h of treatment (Fig. 4E and F). At 4 and 8 μg/mL, no killing was detected. At 16 μg/mL, both analogs led to a ~3 Log_10_ decrease in CFU/mL. Increasing the concentration of AK126 to 32 μg/mL did not yield additional killing compared with 20 μg/mL, while 32 μg/mL of AK127 achieved complete eradication of persister cells.

**Fig 5:**
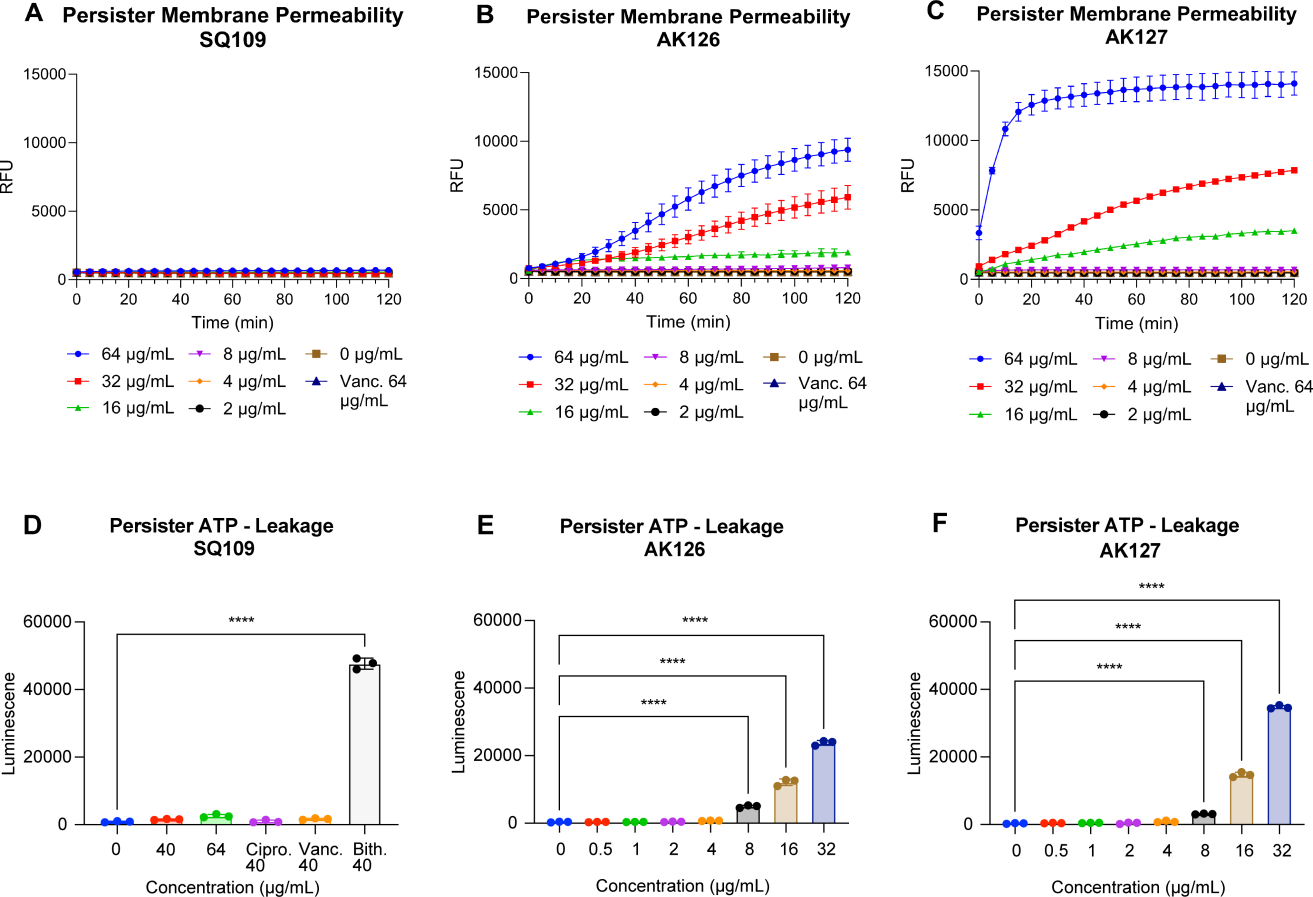
Membrane disruption efficacy of SQ109, AK126, and AK127 against gentamicin-induced *S. aureus* persister cells. (**A–C**) SYTOX green membrane permeability assay after exposure of gentamicin-induced *S. aureus* MW2 persisters with a range of concentrations of SQ109 (**A**), AK126 (**B**), and AK127 (**C**) for 2 h (*n* = 3, replicated thrice). (**D–F**) Leakage of intracellular ATP in gentamicin-induced *S. aureus* MW2 persisters after treatment with SQ109 (at 40 and 64 μg/mL) (**D**), AK126 (0.5–32 μg/mL) (**E**), or AK127 (0.5–32 μg/mL) (**F**) for 1 h (*n* = 3, replicated twice, ****P < 0.0001 by one-way ANOVA followed by Dunnett’s multiple comparison test). Ciprofloxacin (40 μg/mL) and vancomycin (40 μg/mL) were used as antibiotic controls. Bithionol (40 μg/mL) served as a positive control.

We next examined whether AK126 and AK127 perturbed the membrane in MRSA-MW2 persister cells using the membrane-impermeable dye, SYTOX green. Although there was a decrease in the activity of AK126 and AK127 compared to exponential-phase cells, we still noticed an increase in fluorescence after exposure to AK126 and AK127 at 16 μg/mL or higher (Fig. 5B and C). To confirm these observations, we measured the leakage of intracellular ATP following a 1-h treatment across a range of concentrations. Both AK126 and AK127 increased luminescence compared with the untreated control, indicating that the membrane was indeed compromised (Fig. 5E and F). Interestingly, a statistically significant increase in luminescence was observed at concentrations as low as 8 μg/mL and rose in a dose-dependent manner. In contrast, exposure of MRSA-MW2 persister cells to SQ109 at 64 μg/mL over 2 h did not show any increase in fluorescence of SYTOX green (Fig. 5A). Similarly, treating the cells with 64 μg/mL of SQ109 for 1 h did not significantly alter luminescence compared to the control, suggesting that SQ109 cannot damage the membranes of persister cells at this concentration (Fig. 5D).

### Synergistic activity of SQ109 analogs with gentamicin against MRSA persister cells

To further evaluate potential strategies for enhancing the antimicrobial activity of SQ109 and its analogs at lower concentrations, we performed standard checkerboard assays with a panel of antibiotics and calculated the fractional inhibitory concentration index (FICi) (Fig. S4). While no clear synergistic interactions were detected, the combination of SQ109 with gentamicin showed the lowest FICi (0.52) (Fig. S4D).

**Fig 6:**
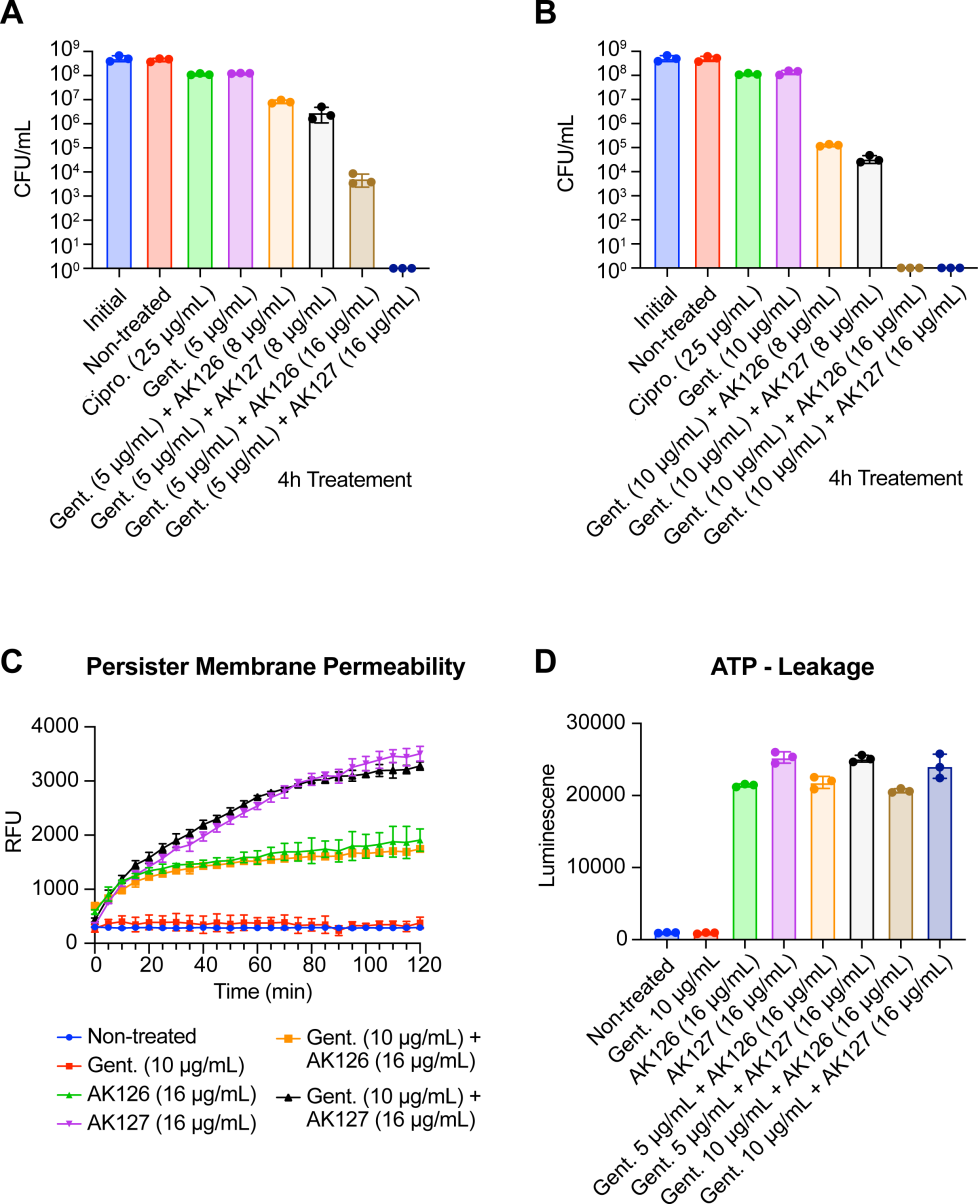
SQ109 analogs potentiate the bactericidal activity of gentamicin against MRSA persister cells. Dose-dependent killing of MRSA-MW2 persister cells after 4 h of treatment with AK126 (8 or 16 μg/mL) or AK127 (8 or 16 μg/mL) in combination with gentamicin at 5 μg/mL (**A**) or 10 μg/mL (**B**). (**C**) Uptake of SYTOX green by MRSA persisters after treatment with gentamicin (10 μg/mL), AK126 (16 μg/mL), AK127 (16 μg/mL), or their combination. (**D**) Leakage of intracellular ATP from MRSA persister cells after treatment for 1 h with gentamicin (10 μg/mL), AK126 (16 μg/mL), AK127 (16 μg/mL), or their combination (replicated thrice).

Given that AK126 and AK127 were able to perturb the bacterial membrane of MRSA persisters, we hypothesized that they might also promote the uptake of aminoglycosides. To test this hypothesis, we performed time-kill assays. Gentamicin in combination with either AK126 or AK127 led to dose-dependent eradication of MRSA persister cells (Fig. 6A and B). Combination of 5 μg/mL of gentamicin (5× MIC) and 16 μg/mL of AK126 (8× MIC) showed an additional 2 Log_10_ reduction in CFU/mL compared to AK126 alone (Fig. 4E), while a combination of 10 μg/mL of gentamicin (10× MIC) and 16 μg/mL of AK126 completely eradicated ~9 × 10^8^ persister cells within 4 h of treatment. Notably, the combination of just 16 μg/mL of AK127 (4× MIC) with 5 μg/mL of gentamicin reduced the number of persister cells below the limit of detection. By comparison, gentamicin and ciprofloxacin alone at 10× MIC resulted in only a ~0.5 Log_10_ reduction in CFU/mL. Since the combination of AK126 and AK127 with gentamicin led to a decrease of ≥2 Log_10_ in CFU/mL, these results demonstrate a synergistic killing effect against MRSA persisters.

We next proceeded to explore whether gentamicin enhances the membrane activity of AK126 and AK127. Interestingly, the addition of 10 μg/mL of gentamicin did not promote the permeability of the MRSA persister membrane (Fig. 6C). Similarly, the combined treatment of gentamicin with the analogs did not trigger additional ATP leakage (Fig. 6D).

### Antibiofilm activity of SQ109 analogs

**Fig 7:**
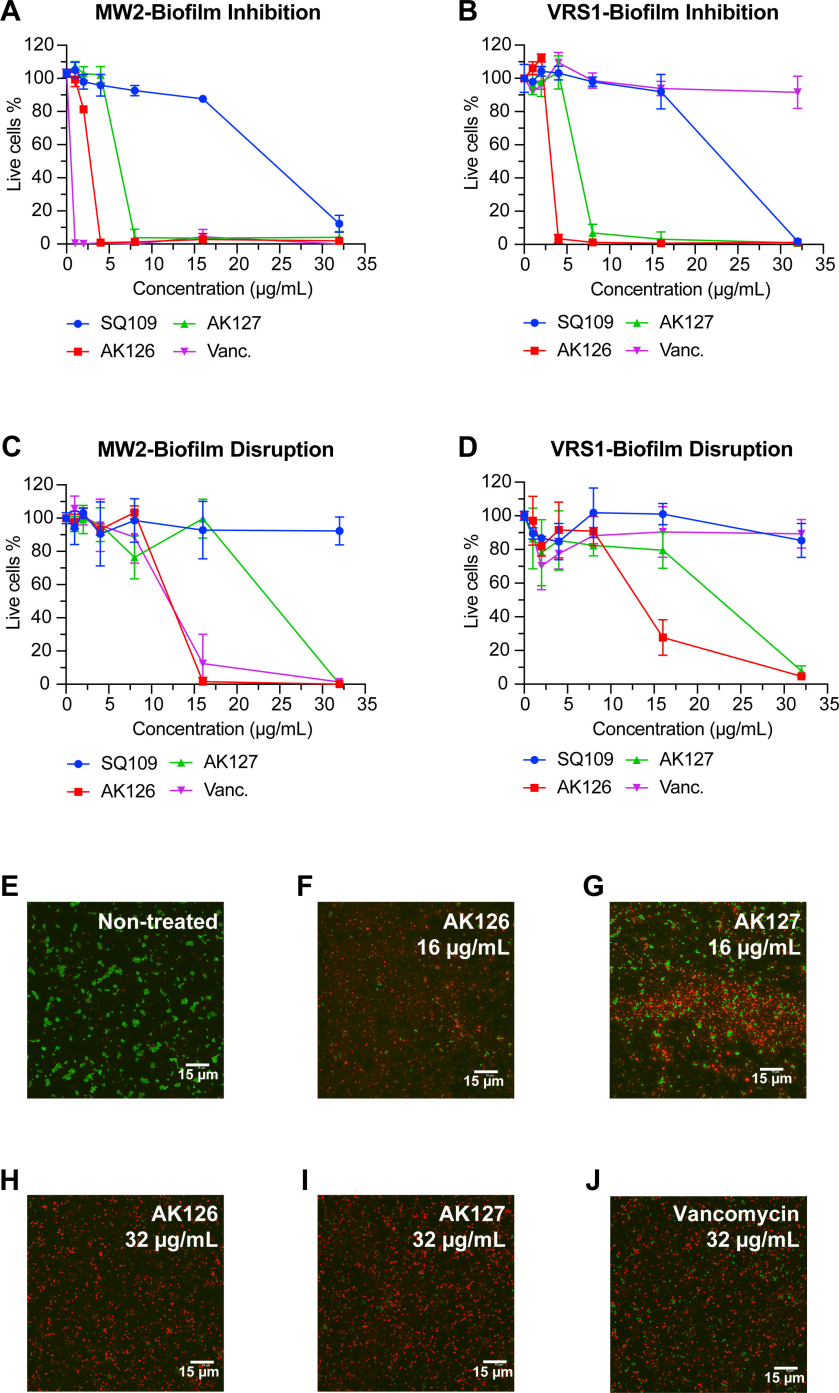
Antibiofilm activity of AK126 and AK127. (**A, B**) Inhibition of *S. aureus* MW2 (**A**) and VRS1 (**B**) biofilm formation assessed using live-cell viability (XTT assay) after 24 h of treatment (*n* = 3, replicated thrice). (**C, D**) Disruption of *S. aureus* MW2 (**C**) and VRS1 (**D**) 24-h biofilms, assessed using live-cell viability (XTT assay) after another 24 h of treatment (*n* = 3, replicated thrice). (**E–J**) Confocal microscopic images of the *S. aureus* MW2 24 h mature biofilm (**E**) and after exposure to 16 μg/mL of AK126 (**F**) or AK127 (**G**) and 32 μg/mL of AK126 (**H**), AK127 (**I**), or vancomycin (**J**) for 18 h (replicated twice).

To further explore the anti-staphylococcal potential of AK126 and AK127, we evaluated their effect on *S. aureus* biofilms. Initially, we tested whether AK126 and AK127 could inhibit biofilm formation in MRSA-MW2 and VRSA-VRS1 strains (Fig. 7A and B). During early biofilm formation, AK126 at 4 μg/mL eradicated 99% of both MRSA-MW2 and VRSA-VRS1 cells, while AK127 at 8 μg/mL produced a similar outcome. In comparison, SQ109 killed 88% of MRSA-MW2 and 99% of VRSA-VRS1 cells at 32 μg/mL. Vancomycin, which was included as an antibiotic control, eliminated 99% of MRSA-MW2 cells at 1 μg/mL, but did not reduce the number of live VRSA-VRS1 cells.

We then investigated whether AK126 and AK127 could disrupt mature *S. aureus* biofilms established after 24 h of growth (Fig. 7C and D; Fig. S5 and S6). AK126 at 16 μg/mL eradicated 98.5% of live MRSA-MW2 cells and 72.2% of VRSA-VRS1 cells within these mature biofilms. By comparison, vancomycin at 16 μg/mL eliminated 87.6% of live MRSA-MW2 cells but had no effect on VRSA-VRS1 biofilms. AK127 at 32 μg/mL reduced the live cell counts by 99.0% in MRSA-MW2 biofilms and by 92.2% in VRSA-VRS1 biofilms. In contrast, SQ109 at 32 μg/mL only lowered live cell counts by 7.7% in MRSA-MW2 biofilm and by 14.6% in VRSA-VRS1 biofilm.

To visualize how AK126 and AK127 affect *S. aureus* biofilms, we employed confocal microscopy (Fig. 7E through J). We exposed 24-h established MRSA-MW2 biofilms overnight to 16 or 32 μg/mL of either AK126 or AK127 and then stained them with SYTO9 (live-cell indicator) and PI (dead-cell indicator). Untreated biofilms appeared predominantly green, reflecting the majority of live cells. Treatment with 16 μg/mL of AK127 resulted in only a partial effect, with an approximate 50% reduction in live cells. In contrast, biofilms exposed to AK126 at both 16 μg/mL and 32 μg/mL, as well as AK127 or vancomycin (positive control) at 32 μg/mL, appeared predominantly red, consistent with our ΧΤΤ—cell viability data (Fig. 7C; Fig. S5).

### Toxicity of SQ109 analogs

**Fig 8:**
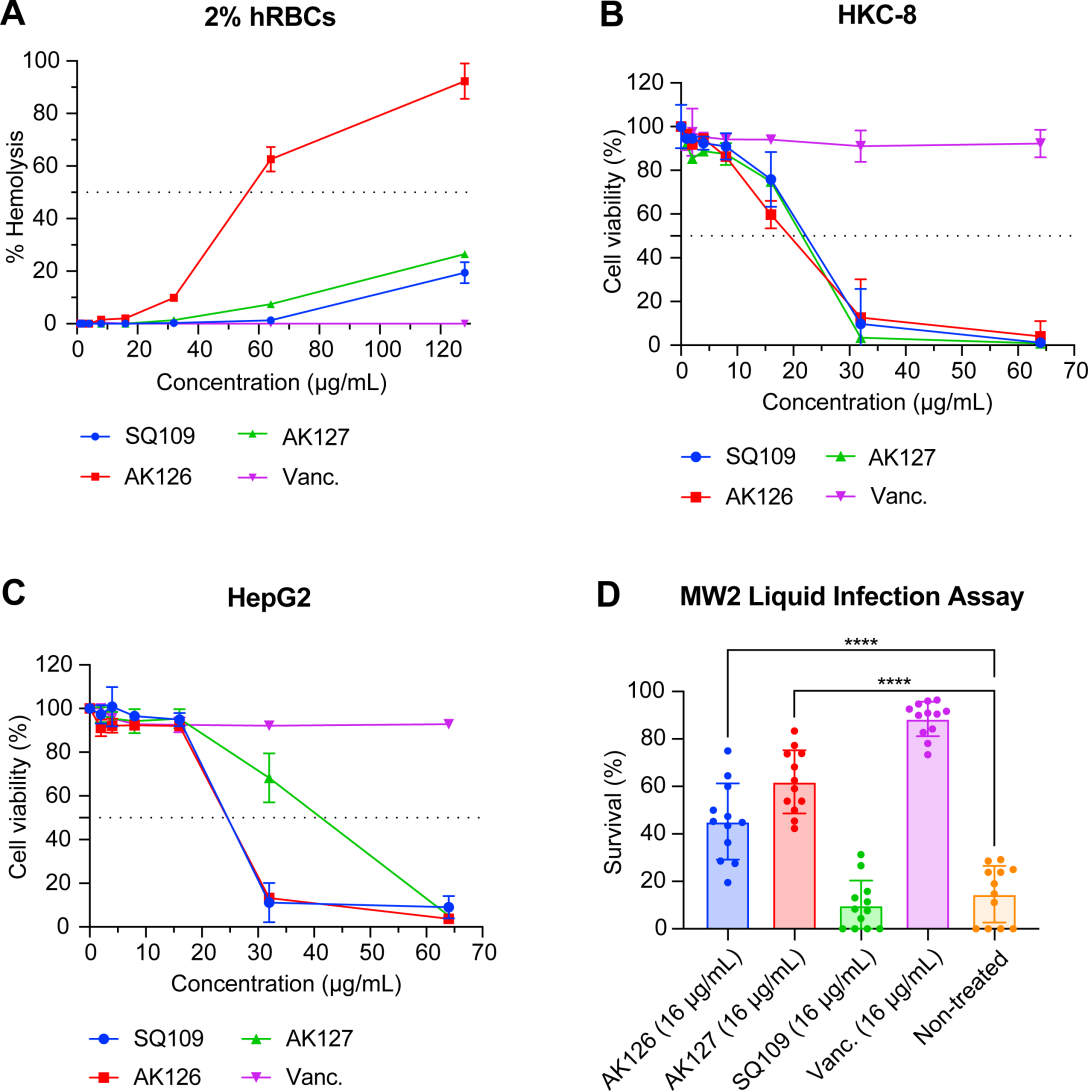
Evaluation of cytotoxic potential and *in vivo* activity of AK126 and AK127. (**A**) Hemolytic activity of SQ109, AK126, and AK127 against 2% hRBCs after exposure for 1 h at various concentrations (*n* = 3, replicated thrice). (**B–C**) Viability of HKC-8 (**B**) and HepG2 cells (**C**) after exposure to various concentrations of SQ109, AK126, or AK127 for 24 h (*n* = 3, replicated thrice). (**D**) Survival of *C. elegans* infected with MRSA-MW2 in the presence of AK126 and AK127 at 16 μg/mL (*n* = 12, replicated twice, ****P < 0.0001 by one-way ANOVA followed by Dunnett’s multiple comparison test). DMSO served as the negative control, and vancomycin at 16 μg/mL served as the positive control. SQ109 at 16 μg/mL was included for comparison.

We evaluated the toxicity of analogs AK126 and AK127 against various mammalian cell types. We first tested their effect on hRBCs (Fig. 8A). Treating 2% hRBCs with AK126 for 1 h resulted in a median hemolytic concentration (HC_50_) of 56.5 μg/mL, translating to a selectivity index (HC_50_/MIC) of 28.1 (Table S1). In contrast, treating hRBCs with AK127 or SQ109 for 1 h caused less than 30% hemolysis at 128 μg/mL.

Next, we examined the toxicity of AK126 and AK127 against human kidney cortex (HKC-8) and human hepatocellular carcinoma (HepG2) cells (Fig. 8B and C; Table S3). A 24-h exposure to AK126 yielded median lethal concentrations (LC_50_) of 19.3 μg/mL for HKC-8 cells and 24.5 μg/mL for HepG2 cells, corresponding to a therapeutic index (LC_50_/MIC) of 9.6 and 12.2, respectively. Similarly, a 24-h exposure to AK127 produced LC_50_ values of 21.5 μg/mL for HKC-8 cells and 41.2 μg/mL for HepG2, resulting in therapeutic indexes of approximately 5 and 10, respectively. Although treating these cell lines with SQ109 showed similar LC_50_ values, SQ109’s higher MIC (16 μg/mL) led to a low therapeutic index (Table S3). Together, these results indicate that analogs AK126 and AK127 exhibit improved selectivity toward bacterial membranes over mammalian membranes.

To evaluate whether we could further improve host cell toxicity, we developed DMPC liposomal formulations of AK126 and AK127. Encapsulation improved the toxicity profiles of both analogs (Fig. S7; Table S4). The liposomal form of AK126 prevented hemolysis of human RBCs at concentrations as high as 128 μg/mL, while the LC₅₀ increased from 19.3 to 33.1 μg/mL in HKC-8 cells and from 24.5 to 29.0 μg/mL in HepG2 cells. Similarly, liposomal AK127 prevented hemolysis (>128 µg/mL) and improved the profile in HKC-8 cells (LC₅₀: 29.8 vs. 21.5 μg/mL).

### *In vivo* efficacy of SQ109 and analogs AK126 and AK127

To further confirm the potency of analogs AK126 and AK127 *in vivo*, we employed a *C. elegans* infection model; for comparison, we also included SQ109 (Fig. 8D). Treatment with analogs AK126 and AK127 significantly improved survival compared with the DMSO-treated negative control group (*P* < 0.0001). AK127 (at 16 μg/mL) demonstrated higher efficacy than analog AK126 (at 16 μg/mL) with mean survival rates (±SD) at 62.0% (±13.3) and 45.2% (±16.1), respectively. However, both were less effective than vancomycin (at 16 μg/mL), which resulted in a mean survival rate (±SD) of 88.4% (±7.3). Treatment with SQ109 (at 16 μg/mL) yielded survival rates similar to the DMSO control at 9.0% (±11.6) and 19.9% (±4.0), respectively.

## DISCUSSION

The increasing emergence of antibiotic-resistant MRSA strains poses a significant public health challenge (3). Drug repurposing has emerged as a valuable strategy for identifying antimicrobial agents with favorable properties while mitigating the risks associated with drug development (65). In this study, we demonstrated that the antitubercular drug SQ109 and two of its ethylenediamine analogs, AK126 and AK127, exhibit potent activity against MRSA by targeting the bacterial membrane. The analogs exhibited stronger bactericidal activity, reduced resistance development, and effectively eliminated persister cells, both alone and synergistically with gentamicin at lower concentrations.

SQ109 is an antibacterial agent that initially gained attention for its activity against *M. tuberculosis* and has since shown efficacy against other bacterial (17, 24, 26) and fungal (22) pathogens. Against *M. tuberculosis*, SQ109 is proposed to inhibit the Mmpl3, a trehalose monomycolate transporter essential for cell wall biosynthesis (66), and to disrupt the PMF by functioning as an uncoupler (17–20). In pathogens lacking the Mmpl3, such as *B. subtilis*, its activity is instead attributed to PMF disruption alone (17, 26). Interestingly, while previous studies (17, 67) reported little to no inhibitory activity of SQ109 against *S. aureus*. We observed moderate activity against MRSA (MIC = 16 µg/mL). Both SQ109 and its two most potent ethylenediamine analogs, AK126 and AK127, induced membrane depolarization and caused severe membrane damage. This dual effect—disrupting both the electrochemical gradient and membrane integrity—suggests a broader and more potent MoA. For example, the uncoupler carbonyl cyanide m-chlorophenylhydrazone (CCCP) is known to collapse the PMF in *S. aureus* cells without causing membrane permeability (68). Consistent with prior work (68) showing that PMF inhibitors can permeabilize membranes only above a certain potency threshold, our findings suggest that SQ109 and its ethylenediamine analogs, AK126 and AK127, demonstrate sufficient potency to exceed this threshold.

Structure–activity relationship analysis of the SQ109 analogs that feature an alkyl or aryl substituent at the adamantyl C-2 position revealed that, generally, compounds retaining the ethylenediamine scaffold exhibited either comparable or enhanced antimicrobial activity. Meanwhile, replacement with an aminoamide scaffold led to a decrease in activity in most analogs. These findings are consistent with observations from our previous study on other pathogens (25, 26), further supporting the importance of the ethylenediamine scaffold in maintaining the antimicrobial properties. Additionally, we found that increasing the size of substituents at C-2 enhances the antimicrobial potency, at least to a certain extent. Notably, the ethylenediamine analogs AK126 and AK127, which feature the bulky hydrophobic benzyl and phenyl substituent, respectively, exhibited enhanced membrane targeting and antimicrobial activity against MRSA cells. These observations are in line with our previous work, where ethylenediamine analogs bearing bulky substituents showed increased antimicrobial potency against the gram-positive *B. subtilis,* but not against other pathogens (26). Similarly, Martin et al. (69) reported that the bulky isopropyl benzene group was critical for the membrane targeting activity of SCH-79797 against *B. subtilis*. Together, these findings suggest that the incorporation of suitable hydrophobic substituents can enhance both antimicrobial potency and membrane targeting activity across different gram-positive pathogens, including *B. subtilis* and MRSA.

All-atom MD modeling provided additional insight into the molecular interactions of SQ109 and its two most potent ethylenediamine analogs with the bacterial membrane. Initial binding of SQ109 and its analogs to the negatively charged phospholipid head groups is mediated by the ethylenediamine group, which is monoprotonated at pH 7 via hydrogen bonding. Following the attachment, the ethylenediamine group loses a proton and is neutralized, while the geranyl group facilitates membrane insertion through strong hydrophobic interactions. Interestingly, compared with SQ109, we found that the addition of bulky substituents at the adamantyl C-2 position might reduce the efficiency by which these molecules are able to bind to the membrane but can enhance membrane penetration due to increased hydrophobic interactions.

Importantly, we also discovered that the ethylenediamine AK126 and AK127 exhibited antibiofilm and anti-MRSA persister activity. Biofilm formation and bacterial persistence are two major causes of chronic and recurrent infections (9). Bacterial persistence is a transient, metabolically inactive state that enables cells to survive antibiotics that target cellular growth processes (70). MRSA persisters can be induced during the stationary phase, during biofilm formation, or under stress conditions in the presence of antibiotics (71, 72). Interestingly, although persister cells are characterized by a reduced PMF (9, 73), they maintain adequate membrane potential to remain viable in this dormant state (74). While in *M. tuberculosis,* disruption of the PMF is highly bactericidal in both active and persister cells (75); in our previous work (33), we demonstrated that this is not the case for MRSA persisters. Ionophores such as valinomycin, nigericin, and monensin, despite being potent PMF inhibitors, failed to induce membrane permeability or reduce the number of MRSA persister populations at concentrations up to 64 μg/mL (33). Similarly, the lipopeptide daptomycin, which also disrupts the PMF, has been reported to lack membrane-permeabilizing activity at similar concentrations (31, 76). Interestingly, both AK126 and AK127 were able to induce leakage of intracellular ATP at sub-lethal concentrations, while the reduction in CFU/mL correlated with the uptake of the SYTOX green dye. These findings indicate that extensive membrane permeability is required for the elimination of MRSA persister cells.

Furthermore, both analogs AK126 and AK127 exhibited synergism with gentamicin in eliminating persister cells. While aminoglycosides are highly effective against growing bacteria, their uptake depends on the PMF (77, 78). Several studies (32, 33, 63, 73, 79) have shown that combining membrane targeting compounds with aminoglycosides can potentiate their bactericidal activity against persister cells and allow the use of lower concentrations, thereby minimizing potential toxicity. This synergistic killing can be attributed to sufficient membrane perturbation to permit gentamicin uptake (33, 80). In some cases (32, 81), the combination of membrane-targeting agents with gentamicin has been reported to accelerate membrane permeability; however, we did not observe this effect with AK126 and AK127. Nevertheless, these results highlight the potential of SQ109 analogs for the treatment of persistent *S. aureus* infection.

Despite the many advantages of membrane-acting antimicrobials, such as anti-persister activity and reduced susceptibility to resistance development, they are often linked to toxicity toward mammalian cells (14, 82). In some instances, like the membrane-acting lipopeptides daptomycin and telavancin, these hurdles can be overcome with changes in dosing regimens (82). In our study, AK126 and AK127 exhibited higher selectivity toward MRSA cells over mammalian cells, as evidenced by the increase in MICs in the presence of PG but not in the presence of zwitterionic lipids, and by their HL₅₀ and LC₅₀ values in human cells, both of which were higher than the concentrations associated with bacterial killing. Moreover, we developed DMPC liposomal formulations of AK126 and AK127, which improved their toxicity profiles compared to the free compounds, suggesting additional opportunities to further improve safety and therapeutic potential.

Overall, this study is the first to comprehensively evaluate the antimicrobial activity of SQ109 and its membrane targeting MoA against MRSA. In addition, we identified two analogs, AK126 and AK127, as promising lead compounds with enhanced antimicrobial potency over the parent molecule. Both analogs were effective in reducing MRSA persister cell populations and disrupting *S. aureus* biofilms while maintaining relatively low cytotoxicity relative to SQ109. Our findings indicate that while PMF dissipation occurs as part of their activity, bactericidal effects against MRSA persisters are most strongly associated with extensive membrane permeability. Importantly, we also found that AK126 and AK127 synergize with gentamicin against persister cells, suggesting their potential use as adjuvants to lower the effective dose of gentamicin. Such combinations could not only enhance efficacy against persistent infections but also help mitigate gentamicin-associated toxicity. Although AK126 and AK127 showed a preference for anionic lipids such as PG, cytotoxicity remains an important consideration for any membrane targeting agent. Nevertheless, the strong antimicrobial and anti-persister activity of AK126 and AK127 highlights the potential of this scaffold as a basis for further optimization. Future work aimed at structural refinement and advanced delivery approaches, including liposomal formulations, could enhance bacterial selectivity while mitigating toxicity, thereby improving their overall therapeutic potential.

## SUPPLEMENTAL MATERIAL

Supplemental material aac.01545-25-s0001.docx Tables S1 to S4; Supplemental methods; Fig. S1 to S7.

ASM does not own the copyrights to Supplemental Material that may be linked to, or accessed through, an article. The authors have granted ASM a non-exclusive, world-wide license to publish the Supplemental Material files. Please contact the corresponding author directly for reuse.
