# Supplementary material for: Ιdentification of SQ109 analogs with enhanced antimicrobial activity against methicillin-resistant Staphylococcus aureus
Source: Antimicrob Agents Chemother. 2026 Feb 18;70(4):e01545-25. doi: 10.1128/aac.01545-25 (PMC13041356; doi:10.1128/aac.01545-25)
Supplement: Supplemental material — Tables S1 to S4; Supplemental methods; Fig. S1 to S7. [file aac.01545-25-s0001.docx]

**Supplementary Materials (SI)**

**Ιdentification of SQ109 Analogs with Enhanced Antimicrobial Activity Against Methicillin-Resistant *Staphylococcus aureus***

Charilaos Dellis^1,2^, George Laros^2^, Kyriakos Georgiou^2^, Liyang Zhang^1^, Lewis Oscar Felix^1^, Nikolas Naziris^3^, Narchonai Ganesan^1^, Jianhua Gu ^4^, Marianna Stampolaki^2^, Costas Demetzos^3^, Ioannis P. Papanastasiou^2^, Biswajit Mishra^1^, Antonios Kolocouris^2*^, Eleftherios Mylonakis^1*^

**Supplementary Tables**

**Table S1.** Physicochemical properties of all nanosystems on the day of preparation

| **System** | **Dh^1^ (nm)** | **PDI^2^** | **ζ-pot^3^ (mV)** |
| --- | --- | --- | --- |
| DMPC | 102.4 ± 3.7 | 0.409 ± 0.016 | 5.2 ± 0.2 |
| DMPC:AK126 | 86.2 ± 1.0 | 0.464 ± 0.005 | 19.3 ± 4.4 |
| DMPC:AK127 | 53.0 ± 1.2 | 0.342 ± 0.013 | 31.6 ± 0.3 |

^1^Dh: hydrodynamic diameter, ^2^PDI: polydispersity index, ^3^ζ-pot: zeta potential

**Table S2.** Relative membrane positioning and maximum compound density of SQ109 and its analogs during molecular dynamics (MD) simulations.

|  | | **SQ109** | **AK126** | **AK127** | **AK121** |
| --- | --- | --- | --- | --- | --- |
| **Replicate 1** | Relative Distance from the membrane center (Å) | 13.37 | 10.88 | 10.95 | 13.32 |
|  | Maximum Compound Density (kg m^-3^) | 7.23 | 7.87 | 9.40 | 10.30 |
| **Replicate 2** | Relative Distance from the membrane center (Å) | 10.96 | 8.49 | 10.91 | 13.34 |
|  | Maximum Compound Density (kg m^-3^) | 8.31 | 6.33 | 10.46 | 10.74 |
| **Average** | Relative Distance from the membrane center (Å) | 12.16 | 9.69 | 10.93 | 13.33 |
|  | Maximum Compound Density (kg m^-3^) | 7.77 | 7.10 | 9.93 | 10.52 |

**Table S3.** Evaluation of cytotoxic potential of SQ109, AK126 and AK127.

| **Cell line** | **SQ109** -HC_50_/LC_50_ (μg/ml) | Selectivety/Therapeutic Index (**SQ109**) | **AK126** -HC_50_/LC_50_ (μg/ml) | Selectivety/Therapeutic Index  (**AK126**) | **AK127** -HC_50_/LC_50_ (μg/ml) | Selectivety/Therapeutic Index  (**AK127**) |
| --- | --- | --- | --- | --- | --- | --- |
| Human Red blood cells | >128 | >8 | 56.5 | 28.1 | >128 | >32 |
| HKC-8 | 22.2 | 1.4 | 19.3 | 9.6 | 21.5 | 5.4 |
| HepG2 | 24.5 | 1.5 | 24.5 | 12.2 | 41.2 | 10.3 |

**Table S4.** Cytotoxic profile of AK126 and AK127 in DMPC liposomes.

| **Cell line** | **AK126 : DMPC** HC_50_/LC_50_ (μg/ml) | **AK127 : DMPC** HC_50_/LC_50_ (μg/ml) |
| --- | --- | --- |
| Human Red blood cells | >128 | >128 |
| HKC-8 | 33.1 | 29.8 |
| HepG2 | 29.0 | 30.4 |

**Methods**

**Synthesis of SQ109 analogs**

Analogs were synthesized via a convergent strategy designed to optimize yield and enable structural diversification. Geranylamine was synthesized by brominating geraniol with phosphorus tribromide (PBr_3_) in dry ether at -5 °C, yielding 1-geranyl bromide. The resulting bromide was then treated with phthalimide in the presence of potassium carbonate (K_2_CO_3_) under reflux in dry tetrahydrofuran (THF) for 24 h, affording N-(1-geranyl)phthalimide in 88% yield. Subsequent hydrazinolysis of the N-geranylphthalimide in refluxing ethanol for 6 h provided geranylamine in 81% yield. Geranylamine was then acylated with bromoacetyl chloride (BrCOCH_2_Cl) and K_2_CO_3_ in dichloromethane (DCM) at room temperature for 24 h, yielding the corresponding bromoacetamide in 91% yield (Sup. Fig. 1). Adamantylamines (4a-h) were synthesized from the corresponding adamantyl alcohols (2a-h), which were obtained by reacting 2-adamantanone (1a-h) with lithium or Grignard reagents. The resulting tertiary alcohols were converted to azides (3a-h), which were subsequently reduced with lithium aluminum hydride (LiAlH_4_) in dry ether at room temperature for 24 h, yielding the desired amines (4a-h). These adamantylamines were subsequently coupled with the bromoacetamide to generate the corresponding aminoamide analogs (5a-h). To obtain the ethylenediamine analogs (6a-e), a key reduction step was performed using a combination of trimethyl chlorosilane (Me_3_SiCl) and LiAlH_4_ in dry DCM at 0 to 5 °C under argon atmosphere for 2.5 h followed by treatment with 10% NaOH at 0 °C (Sup. Fig. 2).

**Supplementary Figures**

**Supplementary Figure 1**

**Sup. Fig. 1 Synthesis of geranylamine. Reagents and conditions:** (a) PBr_3_, dry Et_2_O, -5 °C, 3 h, (98%); (b) phthalamide, K_2_CO_3_, dry THF, 70 °C, overnight; (c) H_2_NNH_2_.H_2_O, EtOH, 80 °C, 6 h, (91%); (d) bromoacetyl chloride, K_2_CO_3_, DCM, 0 °C to rt, 24 h, (86%).**Supplementary Figure 2**

**Sup. Fig. 2 Synthesis of SQ109 derivatives (8a-h,9a-h). Reagents and conditions:** (a) n-BuLi, anhydrous THF, -80 °C, 2 h or Mg, dry ether, 1 h, gentle reflux, Ar, ii) 2-adamantanone, dry THF, -80 °C, 2.5 h rt, iii) H_2_O, 0 °C or NH_4_Cl (sat.), 0 °C (78-89%); (b) i) NaN_3_, dry DCM, TFA, 0 °C, 24 h, rt, ii) NH_3_ 12%, 0 °C, (67-86%); (c) i) LiAlH_4_, dry Et_2_O, 0 °C to 60 °C, 5 h, ii) rt, 24 h, NaOH 15%, H_2_O, 0 °C, (47-82%); (d) geranyl bromoacetamide, Et_3_N, dry THF, rt, 48 h; (e) i) Me_3_SiCl, LiAlH_4_, DCM (anh.), 0–5 °C, 2.5 h, ii) NaOH 15%, H_2_O, 0 °C, (31-38%).

**Supplementary Figure 3**


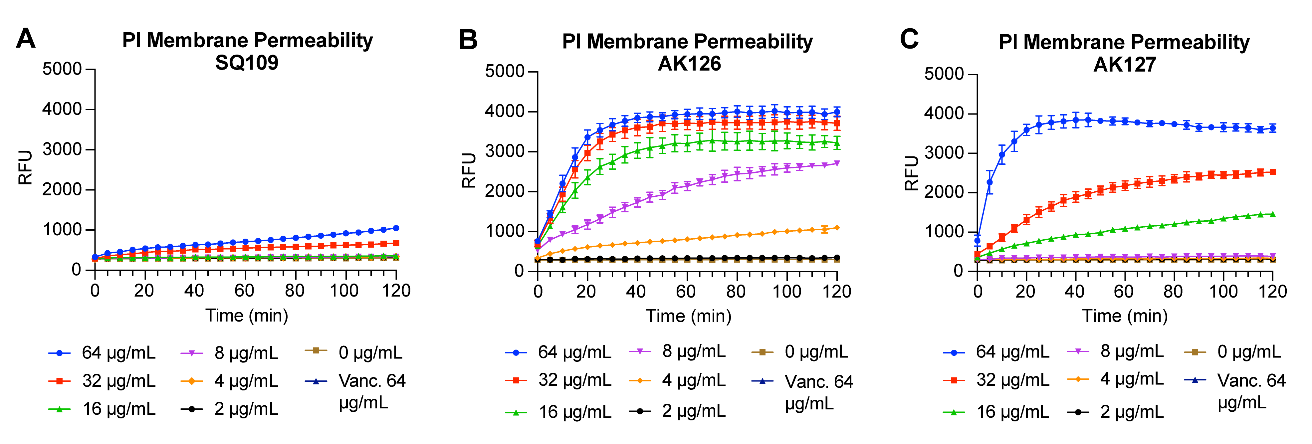


**Sup. Fig. 3 SQ109 and its analogs cause membrane permeability on *S. aureus* exponential phase cells. (A-C)** Uptake of propidium iodide (PI) from *S. aureus* MW2 exponential phase cells after exposure to SQ109 **(A)**, AK126 **(B)** and AK127 **(C)** and monitored for 2 h (n=3, replicated thrice).**Supplementary Figure 4**


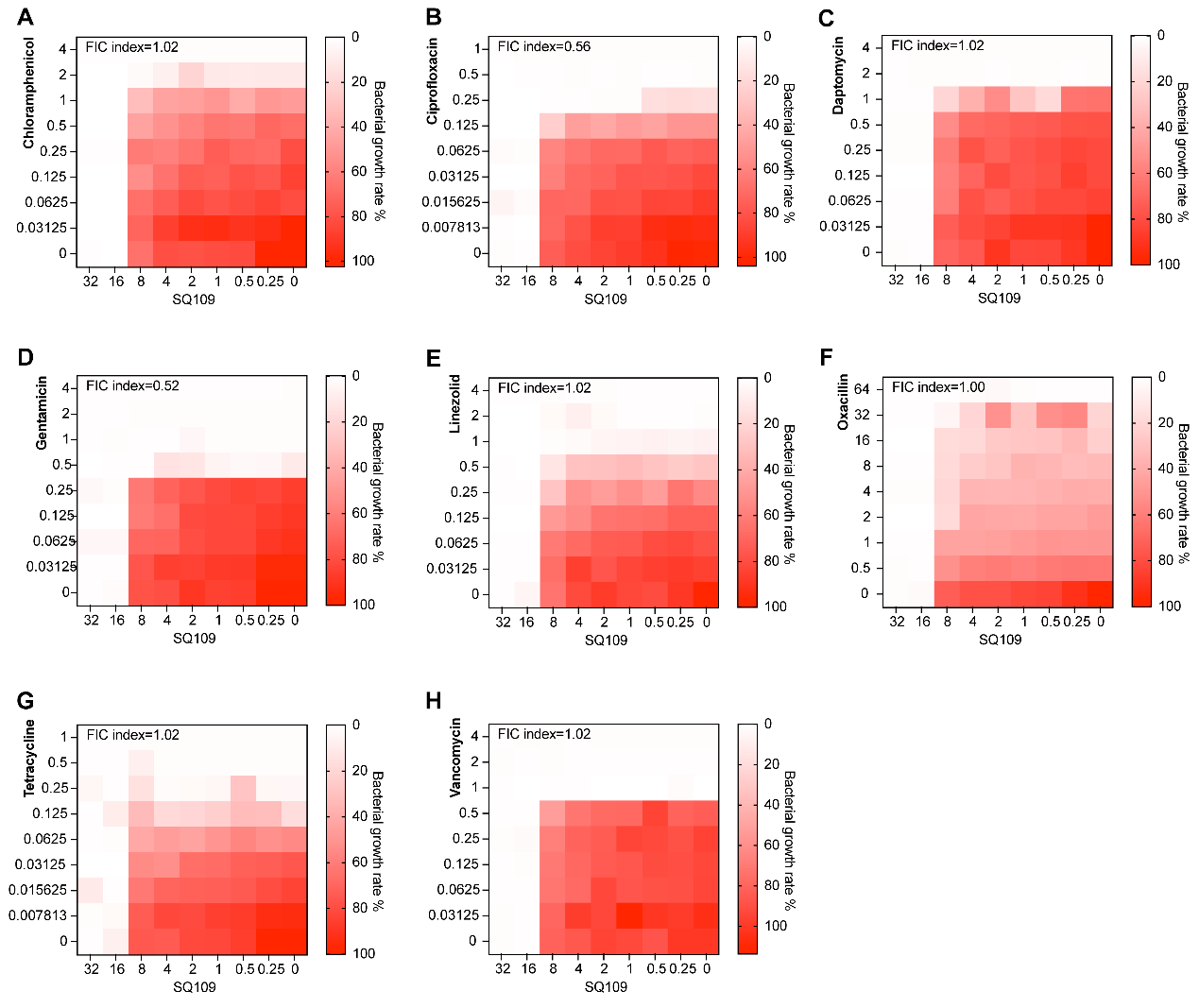


**Sup. Fig. 4 Combination of SQ109 with selected antibiotics against MRSA. (A-H)** Checkerboard microdilution assays of SQ109 combined with chloramphenicol **(A)**, ciprofloxacin **(B)**, daptomycin **(C)**, gentamicin **(D)**, linezolid **(E)**, oxacillin **(F)**, tetracycline **(G)**, and vancomycin **(H)** against MRSA–MW2. Heat maps indicate bacterial growth, with dark red corresponding to higher densities. Synergy was defined as FICi ≤ 0.5. Experiments were replicated twice with similar results.

**Supplementary Figure 5**


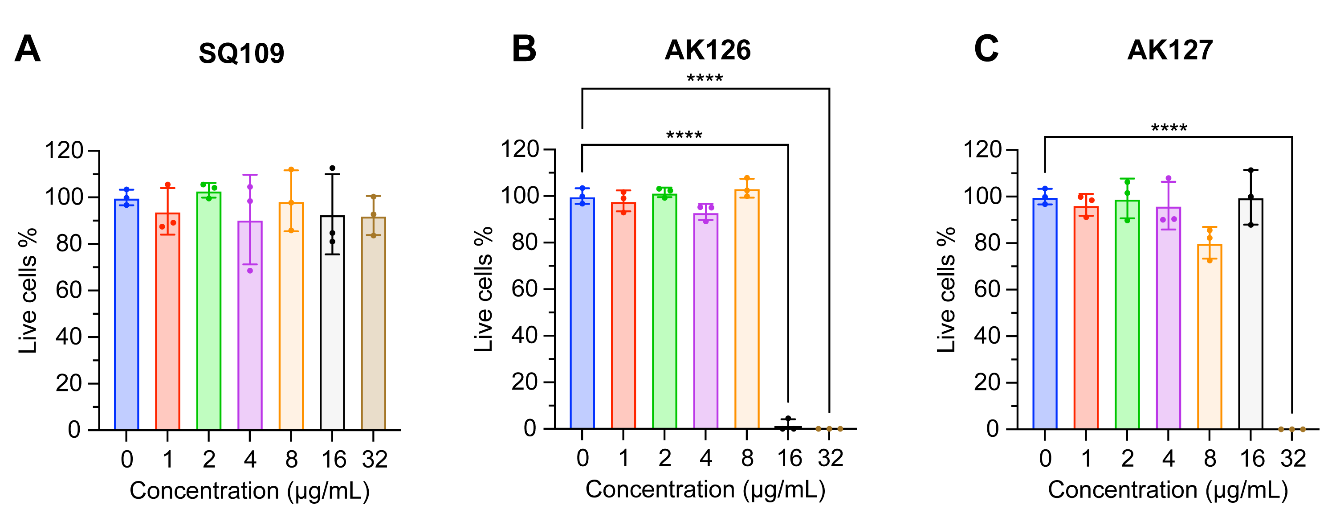


**Sup. Fig. 5 Disruption of *S. aureus* MW2 biofilm.** **(A-C)** Percentage of live MW2 cells present in a 24 h mature biofilm after treatment with SQ109 (1-32 μg/mL) **(A)**, AK126 (1-32 μg/mL) **(B)** or AK127 (1-32 μg/mL) **(C)** (n=3, replicated thrice).

**Supplementary Figure 6**


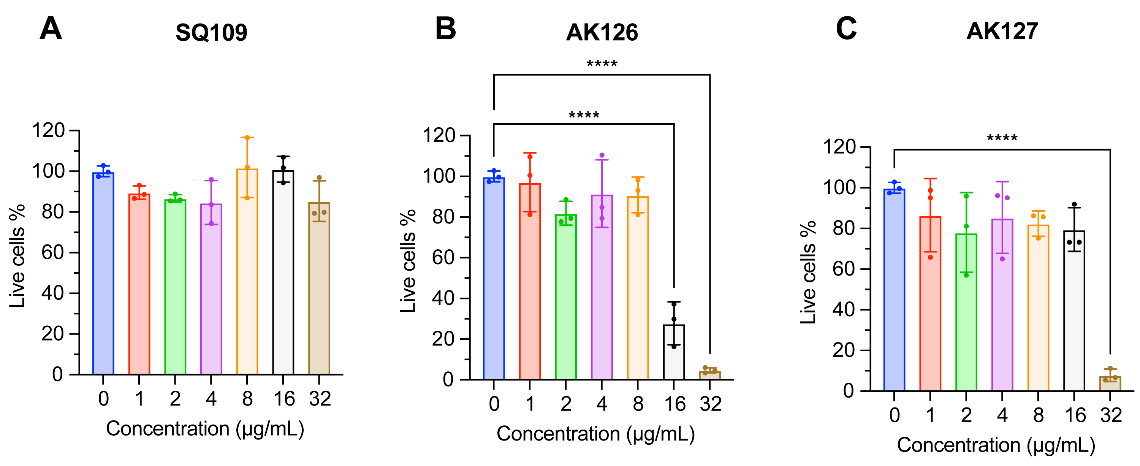


**Sup. Fig. 6 Disruption of *S. aureus* VRS1 biofilm.** **(A-C)** Percentage of live VRS1 cells present in a 24 h mature biofilm after treatment with SQ109 (1-32 μg/mL) **(A)**, AK126 (1-32 μg/mL) **(B)** or AK127 (1-32 μg/mL) **(C)** (n=3, replicated thrice).

**Supplementary Figure 7**


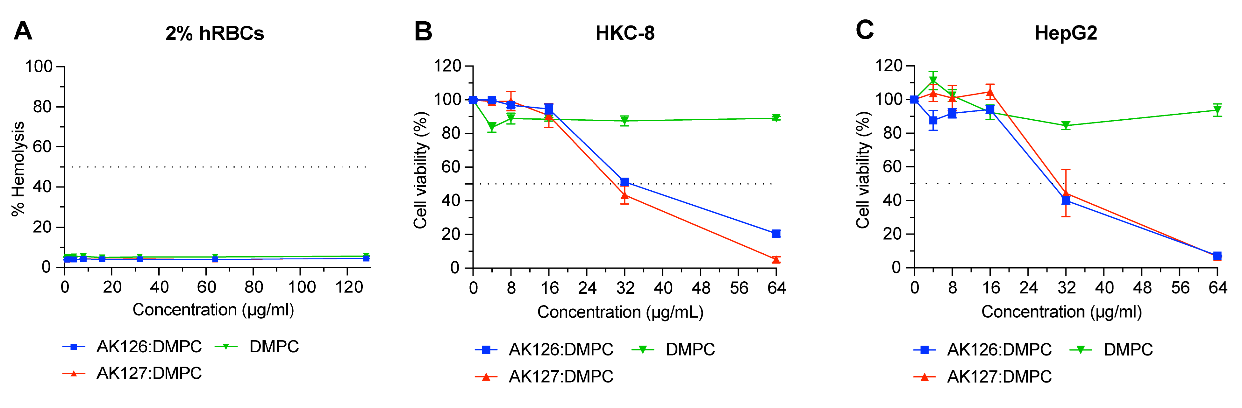


**Sup. Fig. 7** **Cytotoxicity assessment of AK126 and AK127 liposomal formulations. (A)** Hemolytic activity of the liposomal formulations against 2% human RBCs following 1 h exposure at the indicated concentrations. **(B-C)** Viability of HKC-8 **(B)** and HepG2 **(C)** cells after treatment with increasing concentrations of AK126 and AK127 formulated in DMPC liposomes (n=3, performed in triplicate).
